# Minimizing Energy Loss by Designing Multifunctional Solid Additives to Independent Regulation of Donor and Acceptor Layers for Efficient LBL Polymer Solar Cells

**DOI:** 10.1002/advs.202414712

**Published:** 2025-03-20

**Authors:** Junying Wang, Min Deng, Haonan Chen, Wuke Qiu, Yuwei Duan, Chentong Liao, Ruipeng Li, Liyang Yu, Qiang Peng

**Affiliations:** ^1^ College of Materials and Chemistry & Chemical Engineering Chengdu University of Technology Chengdu 610059 P. R. China; ^2^ School of Chemical Engineering and State Key Laboratory of Polymer Materials Engineering Sichuan University Chengdu 610065 P. R. China; ^3^ National Synchrotron Light Source II Brookhaven National Lab Suffolk Upton NY 11973 USA

**Keywords:** energy loss, layer‐by‐layer devices, multifunctional solid additive, polymer solar cells, synergistic effect

## Abstract

Solid additives are crucial in layer‐by‐layer (LBL) polymer solar cells (PSCs). Despite its importance, the simultaneous application of solid additives into both donor and acceptor layers has been largely overlooked. In this work, two multifunctional solid additives are actively designed, and investigated the synergistic effect on both donor and acceptor layers. Incorporating the multifunctional solid additives into the donor layer could effectively enhance the aggregation and molecular stacking of the donor polymer, leading to reduced energy disorder and minimizing Δ*E*_2_. When the multifunctional solid additives are introduced into the acceptor layer, they just play a role in optimizing the morphology, thereby reducing the Δ*E*_3_. Excitedly, the simultaneous addition of the multifunctional solid additives into both donor and acceptor layers produced a synergistic effect for decreasing Δ*E*_2_ and Δ*E*_3_ simultaneously, especially adding SA2, thus enabling an excellent power conversion efficiency (PCE) of 19.95% (certified as 19.68%) with an open‐circuit voltage (*V*_oc_) of 0.921 V, a short circuit current density (*J*_sc_) of 27.08 mA cm^−2^ and a fill factor (FF) of 79.98%. The work highlights the potential of multifunctional solid additives in independently regulating the properties of donor and acceptor layers, which is expected as a promising approach for further developing higher performance PSCs.

## Graphical abstract

The simultaneous incorporation of multifunctional solid additives into both D18 and L8‐BO layers can not only effectively enhance the crystallinity and molecular stacking of D18, but also form an appropriate phase separation morphology, which will reduce Δ*E*_2_ and Δ*E*_3_ and achieve an excellent power conversion efficiency of 19.95% in layer‐by‐layer polymer solar cells.

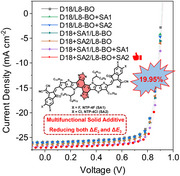

## 1 Introduction

Polymer solar cells (PSCs) have gained attention for the solution‐processed and cost‐effective production of lightweight, large‐area, semi‐transparent, and flexible devices.^[^ ^1^, ^2^, ^3^, ^4^, ^5^ ^]^ Great advances in material design and device engineering have enabled PSCs to achieve ≈20% power conversion efficiency (PCE) by using Y6‐type non‐fullerene small molecular acceptors (NF‐SMAs).^[^ ^6^, ^7^, ^8^ ^]^ Despite those achievements, a significant gap still exists when compared with the low energy loss (*E*_loss_ ≈ 0.4 eV) inorganic solar cells, indicating that a considerable PCE room is expected to be further improved in PSCs if continuously reducing *E*_loss_.^[^ ^9^, ^10^, ^11^, ^12^, ^13^ ^]^ As we know, the *E*_loss_ in PSCs arises from three parts: Δ*E*_1_, Δ*E*_2_, and Δ*E*_3_, where Δ*E*_1_ and Δ*E*_2_ represent radiative recombination energy loss above optical bandgap and below optical bandgap, respectively, and Δ*E*_3_ is the non‐radiative recombination energy loss.^[^ ^14^ ^]^ The Δ*E*_2_ and Δ*E*_3_ are the primary parts of the total *E*_loss_, therefore reducing Δ*E*_2_ and Δ*E*_3_ is crucial for mitigating *E*_loss_. Rational designing the newly efficient chemical structures of photovoltaic materials can reduce Δ*E*_2_ and/or Δ*E*_3_, but is always companied by more complex and labor‐intensive synthesis processes.^[^ ^15^, ^16^, ^17^ ^]^ Apart from the ongoing material synthesis engineering, exploring novel device fabrication processes has become an important and effective method to achieve low *E*_loss_ and high‐performance PSCs in recent years. Among them, the layer‐by‐layer (LBL) deposition process and the use of solid additives are currently the most effective and innovative strategies.^[^ ^18^, ^19^, ^20^, ^21^, ^22^, ^23^ ^]^

The LBL deposition processing method featured a two‐step procedure, which can independently optimize the microstructure of each layer to reduce the impact of complex film‐forming kinetics on the phase separation and crystallization among diverse components. Moreover, this processing method not only spontaneously forms enriched D and A regions at the bottom of the donor layer and the top of the acceptor layer, respectively, achieving higher purity donor and acceptor domains, but also provides sufficient D/A interfaces to promote exciton dissociation, charge transportation, and charge collection, realizing lower *E*_loss_.^[^ ^22^, ^24^, ^25^ ^]^ Coincidentally, solid additives are also widely used to regulate the microscopic structure of the donor or acceptor.^[^ ^26^, ^27^ ^]^ Solid additives that cooperate with LBL deposition processing method can further enhance and expand these benefits, ultimately achieving the optimal morphology with a bicontinuous network to reduce the Δ*E*_2_ and Δ*E*_3_.^[^ ^28^, ^29^ ^]^ However, these solid additives typically only exhibit a single regulatory function and rarely possess dual or even multiple functions to maximize device performance.^[^ ^30^, ^31^, ^32^, ^33^, ^34^ ^]^ Although there have been a few reports of using different solid additives simultaneously to optimize the donor and acceptor layers to achieve the desired morphology, which also has certain limitations, such as increasing the difficulty and time in selecting solid additives, lacking systematic mechanism exploration and in‐depth understanding.^[^ ^29^, ^35^ ^]^ Therefore, actively designing multifunctional solid additives that comprehensively affect the donor layer, acceptor layer and blend morphology in LBL PSCs is not only scientifically inspiring but also a potent strategy to reduce *E*_loss_ and improve efficiency.

**Figure 1:**
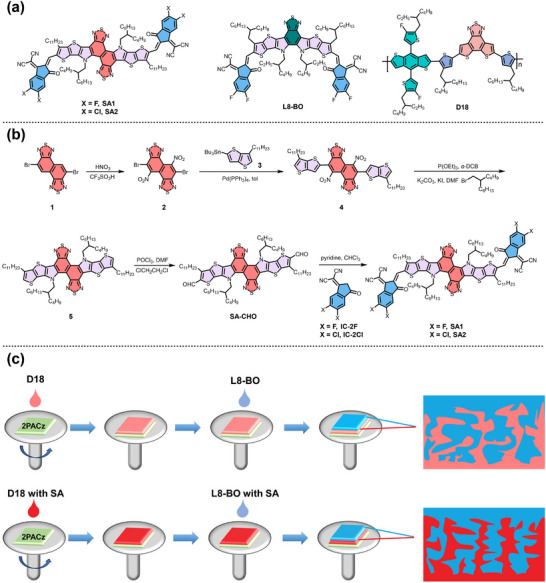
a) Chemical structures of the multifunctional solid additives, L8‐BO and D18. b) Synthetic routes of the multifunctional solid additives. c) Schematic illustration of the active layer formation of LBL PSCs.

In this work, two Y6 derivatives, namely SA1 and SA2 (**Figure** **1**a), were designed and synthesized as high‐performance multifunctional solid additives aiming at efficient LBL PSCs. SA1 and SA2 were prepared by replacing the benzothiadiazole (BT) of Y6^[^ ^36^ ^]^ with naphtho[1,2‐c:5,6‐c]bis[1,2,5]thiadiazole (NT) and terminated with fluorinated or chlorinated end groups, respectively. By adding the multifunctional solid additives into D18 layer, more ordered and tighter *π*‐*π* stacking of D18 could be achieved, which largely suppressed the energy disorder and realized smaller Δ*E*_2_. In addition, when these multifunctional solid additives were added into L8‐BO layer, the molecular stacking of L8‐BO was unaffected, mainly due to its structural similarity ensuring good miscibility. Therefore, in this case, the multifunctional solid additives were just common additives for regulating the morphology with reduced Δ*E*_3_. Excitingly, the simultaneous incorporation of multifunctional solid additives into both D18 and L8‐BO layers could not only effectively enhance the crystallinity and molecular stacking of D18, but also form an appropriate phase separation morphology in the blend films, which could reduce energy disorder and facilitate the charge generation and transportation at the same time. Ultimately, LBL binary PSCs containing multifunctional solid additive of SA2 both in D18 and L8‐BO layers realized the highest PCE of 19.95% with reduced Δ*E*_2_ of 0.055 eV, Δ*E*_3_ of 0.199 eV and total *E*_loss_ of 0.532 eV.

## 2 Results and Discussion

The synthetic routes of the multifunctional solid additives are shown in Figure 1b, and the detailed synthesis procedures are displayed in the Supporting Information. The starting material of compound 1 was synthesized in 6 steps according to a previous report,^[^ ^37^ ^]^ which reacted with fuming nitric acid and trifluoromethanesulfonic acid to provide compound 2 with a yield of 13.28%. Then, compound 2 reacted with compound 3 through the palladium‐catalyzed Stille‐coupling to give compound 4 in 28.31% yield, which was further conducted a bimolecular Cadogan reduction cyclization reaction to produce an intermediate nucleophilic reagent followed by adding 5‐(bromomethyl)undecane as an alkylation reagent to afford compound 5. The yield of compound 5 is extremely low and very unstable, therefore, without any further purification, compound 5 was converted into the key intermediate SA‐CHO through the Vilsmeier–Haack reaction with a yield of 38.26%. The multifunctional solid additives of SA1 and SA2 were finally obtained via the Knoevenagel condensation reaction of SA‐CHO with end groups of IC‐2F and IC‐2Cl with yields of 75.93% and 82.77%, respectively. The chemical structures of intermediates and target molecules were confirmed by ^1^H NMR, ^13^C NMR, and mass spectrometry techniques, and the corresponding spectra were shown in Figures S1–S14 (Supporting Information). SA1 and SA2 displayed good solubility with 24 and 18 mg mL^−1^ in chloroform solution, also with 40 and 35 mg mL^−1^ in chlorobenzene solution, respectively.

The optimized molecular geometries of SA1, SA2, and Y6 were investigated by density functional theory (DFT) with the B3LYP/6‐31G basis set. As shown in Figure S15 (Supporting Information), the optimal molecular configuration of SA1 and SA2 was S‐shape, which was different from the banana shape of Y6. It is evident that when compared with Y6, SA1, and SA2 exhibited a certain degree of twisting in their molecular configuration, indicating the existing distortion, which was most noticeable in the side view where the two molecules showed significant twisting with torsion angles of 151.1° and 149.9° for SA1 and SA2, respectively. Therefore, the conjugated main chains would arrange in almost opposite directions, thus leading to weakened stacking and weaker crystallinity.

The UV–vis–NIR absorption spectra of SA1 and SA2 in dilute chloroform solution and solid‐state thin films were measured. As shown in Figure S16a (Supporting Information), the maximum absorption peaks of SA1 and SA2 were located at 715 and 726 nm with the maximum molar extinction coefficients (*ε*) of 1.23 × 10^5^ and 1.29 × 10^5^ M^−1^ cm^−1^, respectively. From solution to solid‐state film, SA1 and SA2 displayed redshifts of 39 and 53 nm (Figure S16b, Supporting Information). The optical bandgaps (*E*_g_^opt^) of SA1 and SA2 were calculated to be 1.51 and 1.45 eV from the absorption onsets of 823 and 857 nm, respectively. As shown in Figure S16b (Supporting Information), SA1 and SA2 exhibited good complementary absorption with polymer donor D18, which was beneficial for capturing more photons. The energy levels of SA1 and SA2 were investigated by the cyclic voltammetry (CV) method (Figure S16c, Supporting Information), and the corresponding energy diagrams were displayed in Figure S16d (Supporting Information). The highest occupied molecular orbital (HOMO) and lowest unoccupied molecular orbital (LUMO) energy levels were found at −5.80/−3.86 and −5.82/−3.90 eV with the corresponding electronic energy gaps (*E*_g_^cv^) of 1.94 and 1.92 eV for SA1 and SA2, respectively. SA2 demonstrated the deeper HOMO/LUMO energy levels, relating to the empty 3d orbitals of chlorine atoms, which was consistent with the trend observed in the previously reported literature.^[^ ^38^, ^39^ ^]^ The raised LUMO energy level could realize a higher open‐circuit voltage (*V*_oc_) value.

In order to better study the photovoltaic performance of these two NF‐SMAs, PSCs devices were prepared and characterized with a LBL structure of ITO/2‐(9H‐carbazol‐9‐yl) (2PACz)/D18/acceptor/PNDIT‐F3N/Ag. The weight average molecular weight (*M*_w_), number average molecular weight (*M*_n_), and polymer dispersity index (*PDI*) of D18 were calculated to be 148.319, 91.242 kDa, and 1.63 (Figure S17, Supporting Information), respectively. The current density versus voltage (*J*–*V*) curves of the top‐performing devices were shown in Figure S16e (Supporting Information), and the photovoltaic parameters were summarized in Table S1 (Supporting Information). The D18/SA1 devices presented a PCE of 12.72%, with a *V*_oc_ of 0.969 V, a short circuit current density (*J*_sc_) of 20.03 mA cm^−2^ and a fill factor (FF) of 65.55%. The D18/SA2 devices displayed a decreased *V*_oc_ of 0.937 V for their relatively lower LUMO energy level (Figure S16d, Supporting Information). The *J*_sc_ and FF values increased to 21.92 mA cm^−2^ and 69.79%, respectively, which could be attributed to the broader photon response and stronger external quantum efficiency (EQE, Figure S16f, Supporting Information) response. Finally, the D18/SA2 devices exhibited an excellent PCE of 14.33%. At the same time, the device performance of ternary devices based on SA1 and SA2 was also investigated (Figure S18 and Tables S2 and S3, Supporting Information). The ternary devices achieved the highest PCE of 18.42% and 18.58% when adding 6 wt.% SA1 or SA2, respectively. However, if increasing the content of SA1 or SA2, the device performance would decrease sharply.

**Figure 2:**
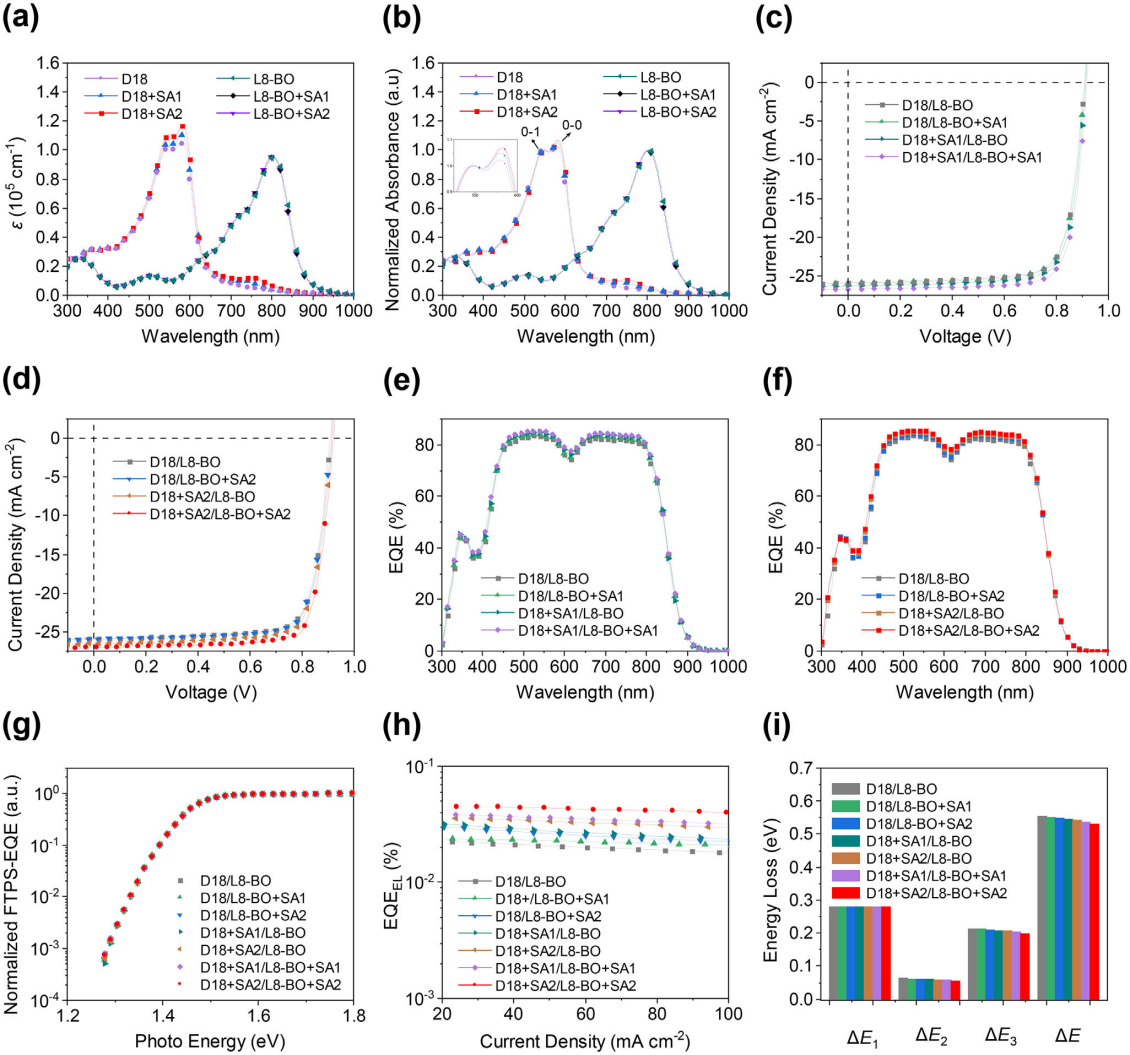
a) UV–vis–NIR absorption spectra of D18 and L8‐BO with or without multifunctional solid additives in solid‐state thin films. b) Normalized UV–vis–NIR absorption spectra of D18 and L8‐BO with or without multifunctional solid additives in solid‐state thin films. c,d) *J*–*V* curves and e,f) EQE curves of the top‐performing devices based on binary blends. g) FTPS‐EQE of the PSCs devices at the absorption onset. h) EQE_EL_ curves of the PSCs. i) Energy loss diagrams of the PSCs.

In order to better investigate the effect of the multifunctional solid additives on D18 and L8‐BO, the UV–vis–NIR absorption spectra of solid‐state thin films were performed. As presented in **Figure** **2**a,b, D18+SA1 (D18 blended with 2% SA1, w/w), D18+SA2 (D18 blended with 2% SA2, w/w) and D18 displayed similar absorption profiles ranging from 300 to 650 nm. The maximum extinction coefficients of D18+SA1 and D18+SA2 were calculated to be 1.10 × 10^5^ and 1.17 × 10^5^ cm^−1^, respectively, which were higher than that of D18 (1.04 × 10^5^ cm^−1^). More importantly, by normalizing the 0‐1 absorption peak, D18+SA1 and D18+SA2 displayed stronger 0‐0 absorption peaks than D18, with the corresponding ratio of 1.05, 1.08, and 1.02 by height of 0‐0/0‐1 (Figure 2b), respectively. The increased extinction coefficient and stronger 0‐0 absorption peak indicated that these multifunctional solid additives could enhance the intermolecular *π*‐*π* interaction and promote the orderly stacking of D18.^[^ ^40^, ^41^ ^]^ It is interesting that D18+SA1 and D18+SA2 also exhibited slightly stronger absorption than D18 in the range of 650–850 nm, which originated from the absorption of SA1 and SA2. On the other hand, L8‐BO+SA1 (L8‐BO blended with 6% SA1, w/w), L8‐BO+SA2 (L8‐BO blended with 6% SA2, w/w) and L8‐BO also exhibited almost identical absorption curves across the entire spectrum with a maximum extinction coefficient of 9.52 × 10^4^ cm^−1^ (Figure 2a), suggesting that the multifunctional solid additives had negligible effect on molecular stacking of L8‐BO. In addition, the same results were confirmed by the grazing‐incidence wide‐angle X‐ray scattering (GIWAXS) measurements. The D18+SA1, D18+SA2, L8‐BO+SA1, and L8‐BO+SA2 thin films remained face‐on stacking orientation (Figure S19, Supporting Information). The D18+SA1 and D18+SA2 thin films displayed tighter lamellar stacking distance (*d*_l_) of 2.19 and 2.16 nm than that of D18 thin film (2.21 nm) (Table S4, Supporting Information), where the (100) peaks were located at 2.87, 2.91 and 2.84 nm^−1^ for D18+SA1, D18+SA2 and D18 thin films along the in‐plane (IP) direction, respectively. Along the out‐of‐plane (OOP) direction, the (010) peaks were found at 16.95, 17.05, and 16.90 nm^−1^ for D18+SA1, D18+SA2, and D18 thin films with a similar *π*‐*π* stacking distance (*d*_π_) of 0.37 nm. Besides, the D18+SA1 and D18+SA2 thin films exhibited crystal coherence lengths (CCL)^[^ ^42^, ^43^ ^]^ of 6.51 and 6.91 nm in the IP direction, as well as 2.60 and 2.77 nm in the OOP direction, which were larger than those of D18 of 6.15 and 2.45 nm, respectively. However, the characteristic crystallization peaks of L8‐BO, L8‐BO+SA1, and L8‐BO+SA2 thin films remained almost unchanged both in the IP and OOP directions, showing almost identical values of *d*_l_, *d*_π_, and CCL. The above results further demonstrated that the addition of multifunctional solid additives could improve the crystallinity of D18 and promote its ordered stacking, while there were almost no interactions between L8‐BO and multifunctional solid additives.

The electrostatic potential (ESP) distributions of D18, L8‐BO, and multifunctional solid additives were calculated by using density functional theory (DFT) with the B3LYP/6‐31G basis set to further investigate the intermolecular interaction. As provided in Figure S20 (Supporting Information), D18 exhibited negative charge distribution in the BDT and DTBT units, and positive charge distribution on alkyl chains and hydrogens of thiophene. However, L8‐BO and multifunctional solid additives predominantly performed positive ESP distribution, particularly in the central core. Consequently, it can be deduced that a strong intermolecular interaction between D18 and the multifunctional solid additives due to the opposite polarity of the charge distributions.^[^ ^44^, ^45^ ^]^ Furthermore, SA1 showed a more positive average ESP of 8.97 kcal mol^−1^ than that of SA2 (8.54 kcal mol^−1^), indicating stronger intermolecular interactions between D18 and SA1, which would cause the polymer to precipitate out from solution rapidly, affecting the overall performance of polymer donor.^[^ ^17^ ^]^ However, benefiting from the relatively weak intermolecular interaction between D18 and SA2, which elongated the time for nucleation and crystal growth to achieve tighter and more ordered molecular stacking.^[^ ^46^, ^47^, ^48^ ^]^ According to the in situ UV–vis measurements (Figure S21, Supporting Information),^[^ ^49^ ^]^ it could be clearly seen from Figure S21a (Supporting Information) that the red and green regions on the left side of the line A were relatively stable, indicating that the region was in the solvent evaporation stage (I). Between line A and line B, a clear downward transition could be observed in the red and green regions with an increase in width, which was the rapid transition from liquid to solid due to the solvent evaporation, known as stage II. On the right side of line B, the red and green regions had stabilized and the width no longer changed, indicating that the film had been completely formed (stage III). Obviously, the D18+SA1 films lasted for a shorter duration time in the solvent evaporation stage (I), indicating stronger intermolecular interactions between D18 and SA1.^[^ ^50^ ^]^ Besides, D18+SA2 films displayed a longer crystallization time of 0.30 s than the D18+SA1 films (0.20 s) in the rapid crystallization stage (II), which contributed to forming a more complete crystal and led to stronger crystallinity.^[^ ^51^ ^]^

The PSCs devices were fabricated with a LBL structure of ITO/ 2PACz/donor/acceptor/PNDIT‐F3N/Ag to study the effect of multifunctional solid additives on the photovoltaic performance. The *J*–*V* curves and the corresponding photovoltaic parameters of the optimal devices containing multifunctional solid additives are shown in Figure 2c,d, and **Table** **1**. The D18/L8‐BO devices presented a PCE of 18.18% with a *V*_oc_ of 0.907 V, a *J*_sc_ of 25.86 mA cm^−2,^ and an FF of 77.51%, which were comparable to the reported values.^[^ ^52^ ^]^ The *V*_oc_, *J*_sc_ and FF values of the D18/L8‐BO+SA1 and D18/L8‐BO+SA2 devices increased mildly to 0.911 and 0.913 V, 25.95 and 26.05 mA cm^−2^, 77.91% and 78.12%, respectively, which were due to that the addition of multifunctional solid additives into L8‐BO layer would regulate the blend film morphology (Figure S22 and Table S5, Supporting Information).^[^ ^38^, ^39^, ^53^ ^]^ Hence, the PCE enhanced to 18.42% and 18.58% for D18/L8‐BO+SA1 and D18/L8‐BO+SA2 devices, respectively. When the multifunctional solid additives were added into D18 layer, the D18+SA1/L8‐BO and D18+SA2/L8‐BO devices presented higher PCEs of 18.82% and 19.04%, respectively, with the raised *V*_oc_, *J*_sc_ and FF values up to 0.914 and 0.916 V, 26.31 and 26.48 mA cm^−2^, 78.26% and 78.50%, respectively, attributing from higher crystallinity and more ordered molecular stacking of D18+SA1 and D18+SA2. What's even more exciting is that the simultaneous addition of multifunctional solid additives to D18 and L8‐BO layers realized unimaginable success, especially the D18+SA2/L8‐BO+SA2 devices achieved an outstanding PCE of 19.95% (certified as 19.68%, Figure S23, Supporting Information) with an increased *V*_oc_ of 0.921 V, an enhanced *J*_sc_ of 27.08 mA cm^−2^ and a remarkable FF of 79.98%. That is one of the highest PCE in LBL binary PSCs at present. Similarly, the devices containing multifunctional solid additive SA1 also exhibited excellent performance with an outstanding PCE of 19.49%, with a *V*_oc_ of 0.919 V, a *J*_sc_ of 26.72 mA cm^−2^ and an FF of 79.37%. The significant improvement in device performance can be attributed to the synergistic effect of the multifunctional solid additives, which not only enhanced crystallinity and promoted ordered molecular stacking of D18, but also optimized the blend film morphology. As shown in Figure 2e,f, all the devices exhibited similar EQE curves in the range of 300–1000 nm. The *J*_sc_^EQE^ values calculated from EQE spectra were 24.93, 25.10, 25.21, 25.29, 25.47, 25.54 and 25.80 mA cm^−2^ for D18/L8‐BO, D18/L8‐BO+SA1, D18/L8‐BO+SA2, D18+SA1/L8‐BO, D18+SA2/L8‐BO, D18+SA1/L8‐BO+SA1 and D18+SA2/L8‐BO+SA2 based devices, respectively, which were consistent with the values from the *J*–*V* curves within 5% error. Moreover, the potential application of SA2 in other systems was also studied (Figure S24 and Tables S6 and S7, Supporting Information). When the multifunctional solid additive SA2 was added into D18/BTP‐eC9 blend film, the PCE enhanced from the control devices of 17.58%–18.15, 18.72 and 19.22% for D18/BTP‐eC9+SA2, D18+SA2/BTP‐eC9 and D18+SA2/BTP‐eC9+SA2 devices, respectively. Similarly, when SA2 was added simultaneously into D18 and BTP‐Cy‐4F layers, where the acceptor BTP‐Cy‐4F was developed by our group,^[^ ^39^ ^]^ the PCE significantly increased from 17.97% of the control devices to 19.47% for the D18+SA2/BTP‐Cy‐4F+SA2 device. The above results indicated that SA2 showed great application potential to enhance the photovoltaic performance in other systems. In addition, compared with other additives of 1,8‐diiodooctane (DIO) and 1‐chloronaphthalene (CN), the devices based on SA1 and SA2 also exhibited better photovoltaic device performance and higher stability (Figures S25 and S26, Tables S8 and S9, Supporting Information).

**Table 1:** Photovoltaic parameters of the LBL devices.

active layer	*V*_oc_ (V)	*J*_sc_ [mA cm^−2^]	*J*_sc_^EQE^ [mA cm^−2^]	FF (%)	PCE (%)
D18/L8‐BO	0.907 (0.905 ± 0.005)	25.86 (25.71 ± 0.22)	24.93	77.51 (77.39 ± 0.32)	18.18 (18.01 ± 0.13)
D18/L8‐BO+SA1	0.911 (0.909 ± 0.004)	25.95 (25.83 ± 0.18)	25.10	77.91 (77.76 ± 0.30)	18.42 (18.25 ± 0.14)
D18/L8‐BO+SA2	0.913 (0.912 ± 0.002)	26.05 (25.89 ± 0.16)	25.21	78.12 (77.79 ± 0.37)	18.58 (18.37 ± 0.15)
D18+SA1/L8‐BO	0.914 (0.912 ± 0.003)	26.31 (26.16 ± 0.17)	25.29	78.26 (78.19 ± 0.09)	18.82 (18.65 ± 0.13)
D18+SA2/L8‐BO	0.916 (0.915 ± 0.004)	26.48 (26.07 ± 0.29)	25.47	78.50 (78.31 ± 0.32)	19.04 (18.68 ± 0.20)
D18+SA1/L8‐BO+SA1	0.919 (0.918 ± 0.002)	26.72 (26.61 ± 0.23)	25.54	79.37 (79.27 ± 0.18)	19.49 (19.37 ± 0.18)
D18+SA2/L8‐BO+SA2	0.921 (0.920 ± 0.004)	27.08 (26.87 ± 0.20)	25.80	79.98 (79.70 ± 0.37)	19.95 (19.62 ± 0.15)

*Note*: The average values and standard deviations were obtained from 10 individual devices.

In addition, we explored the device performance by using PEDOT:PSS as a hole‐transporting layer (Figure S27 and Table S10, Supporting Information). The D18/L8‐BO devices displayed a limited PCE of 17.28%, with a *V*_oc_ of 0.902 V, a *J*_sc_ of 24.91 mA cm^−2,^ and an FF of 76.89%. If adding the multifunctional solid additive into L8‐BO layer, the PCE, *V*_oc_, *J*_sc_, and FF values slightly enhanced to 17.72% and 18.01%, 0.904 and 0.905 V, 25.32 and 25.59 mA cm^−2^, 77.41% and 77.78% for D18/L8‐BO+SA1 and D18/L8‐BO+SA2 devices, respectively. Similarly, when adding the multifunctional solid additive into D18 layer, the related devices achieved the increased PCEs of 18.36% and 18.64%, with *V*_oc_s of 0.908 and 0.910 V, *J*_sc_s of 25.87 and 26.12 mA cm^−2^ and FFs of 78.16% and 78.41% for D18+SA1/L8‐BO and D18+SA2/L8‐BO devices, respectively. If simultaneously incorporating the multifunctional solid additive into both D18 and L8‐BO layers, the devices realized tremendous success with excellent PCEs of 18.98% and 19.26%, with *V*_oc_ values increased to 0.912 and 0.914 V, *J*_sc_ values raised to 26.36 and 26.62 mA cm^−2^ and FF values enhanced to 78.93% and 79.15%, respectively. Compared with the hole‐transporting layer of 2PACz, the device performance based on PEDOT:PSS was slightly inferior, which was due to that 2PACZ could better promote exciton dissociation and charge transfer, thereby achieving enhanced photovoltaic performance.^[^ ^54^ ^]^

The *E*_loss_ measurements were conducted to investigate the origin of high *V*_oc_ and PCE. The calculation of each parameter in *E*_loss_ was provided in the supporting information. All the blend films possessed identical optical bandgaps of 1.457 eV by fitting EQE spectra.^[^ ^55^, ^56^ ^]^ The *E*_loss_ could be separated into three parts:^[^ ^57^ ^]^ i) Δ*E*_1_ = *E*_g_ – *qV*_oc, SQ_, where *V*_oc, SQ_ represents maximum voltage predicted by Shockley–Queisser (SQ) theory; ii) Δ*E*_2_ = *qV*_oc, SQ_ – *qV*_oc, rad_, where *V*_oc, rad_ is the *V*_oc_ without non‐radiative recombination; iii) Δ*E*_3_ = −*kT*(lnEQE_EL_),^[^ ^58^, ^59^ ^]^ where EQE_EL_ is the electroluminescence quantum efficiency. The D18/L8‐BO, D18/L8‐BO +SA1, and D18/L8‐BO+SA2 blend films showed the same *V*_oc, SQ_ of 1.177 V with Δ*E*_1_ of 0.280 eV, the D18+SA1/L8‐BO and D18+SA2/L8‐BO, D18+SA1/L8‐BO+SA1 and D18+SA2/L8‐BO+SA2 blend films showed the same *V*_oc, SQ_ of 1.178 and 1.179 V with Δ*E*_1_ of 0.279 and 0.278 eV (Table S11, Supporting Information). The results indicated that the multifunctional solid additives had negligible impact on Δ*E*_1_. The *V*_oc, rad_ values were observed at 1.115, 1.116, 1.117, 1.119, 1.120, 1.123, and 1.124 V, corresponding to the Δ*E*_2_ values of 0.062, 0.061, 0.060, 0.059, 0.058, 0.056, and 0.055 eV for D18/L8‐BO, D18/L8‐BO+SA1, D18/L8‐BO+SA2, D18+SA1/L8‐BO, D18+SA2/L8‐BO, D18+SA1/L8‐BO+SA1 and D18+SA2/L8‐BO+SA2 blend films, respectively. Actually, the decreased Δ*E*_2_ was associated with the suppressed energy disorder,^[^ ^16^, ^17^ ^]^ which could be quantified by the Urbach energy (*E*_U_). By fitting the Fourier‐transform photocurrent spectroscopy‐external quantum efficiency (FTPS‐EQE) spectra (Figure 2g; Figure S28, Supporting Information), the *E*_U_ values were 24.19, 24.10, 24.05, 21.96, 21.80, 21.45, and 21.27 meV for D18/L8‐BO, D18/L8‐BO+SA1, D18/L8‐BO+SA2, D18+SA1/L8‐BO, D18+SA2/L8‐BO, D18+SA1/L8‐BO+SA1 and D18+SA2/L8‐BO+SA2 devices, respectively. The smaller *E*_U_ represented the lower energy disorder and more ordered molecular stacking,^[^ ^56^, ^60^ ^]^ further confirming that the multifunctional solid additives promoted the molecular stacking to suppress the energy disorder and thus reduce Δ*E*_2_ in LBL devices. In addition, the *E*_U_ values were measured by fitting the tail state of the EQE curves (Figure S29, Supporting Information).^[^ ^61^, ^62^ ^]^ The calculated *E*_U_ values were 25.39, 25.28, 25.23, 22.96, 22.72, 22.32, and 22.20 meV for D18/L8‐BO, D18/L8‐BO+SA1, D18/L8‐BO+SA2, D18+SA1/L8‐BO, D18+SA2/L8‐BO, D18+SA1/L8‐BO+SA1 and D18+SA2/L8‐BO+SA2 devices, respectively. The *E*_U_ values measured by the two methods were within 5% error, indicating that the *E*_U_ values obtained from FTPS‐EQE spectra were accurate. The D18/L8‐BO blend films obtained an EQE_EL_ of 0.025%, while higher EQE_EL_ values of 0.026%, 0.028%, 0.032%, 0.034%, 0.039% and 0.044% were attained for D18/L8‐BO+SA1, D18/L8‐BO+SA2, D18+SA1/L8‐BO, D18+SA2/L8‐BO, D18+SA1/L8‐BO+SA1 and D18+SA2/L8‐BO+SA2 blend films (Figure 2h), respectively. Therefore, D18/L8‐BO+SA1, D18/L8‐BO+SA2, D18+SA1/L8‐BO, D18+SA2/L8‐BO, D18+SA1/L8‐BO+SA1 and D18+SA2/L8‐BO+SA2 blend films realized the suppressed Δ*E*_3_ of 0.211, 0.209, 0.207, 0.205, 0.202 and 0.199 eV than that of D18/L8‐BO of 0.213 eV. Obviously, the use of multifunctional solid additives would be helpful to achieve a more ideal morphology. This value (Δ*E*_3_ = 0.199 eV) is one of the lowest non‐radiative recombination loss values in PSCs reported to date. Finally, the *E*_loss_ values of D18/L8‐BO, D18/L8‐BO+SA1, D18/L8‐BO+SA2, D18+SA1/L8‐BO, D18+SA2/L8‐BO, D18+SA1/L8‐BO+SA1, and D18+SA2/L8‐BO+SA2 blend films were estimated to be 0.555, 0.552, 0.549, 0.545, 0.542, 0.536 and 0.532 eV (Figure 2i), respectively. It was clear that simultaneously adding multifunctional solid additives into D18 and L8‐BO layers would help to achieve smaller Δ*E*_2_, Δ*E*_3_, and *E*_loss_, arising from the synergistic effect of the multifunctional solid additives, which not only enhanced the crystallinity and ordered molecular stacking of D18, but also induced more ideal film morphology with suppressed energy disorder as well as enhanced exciton dissociation and charge transfer properties.

**Figure 3:**
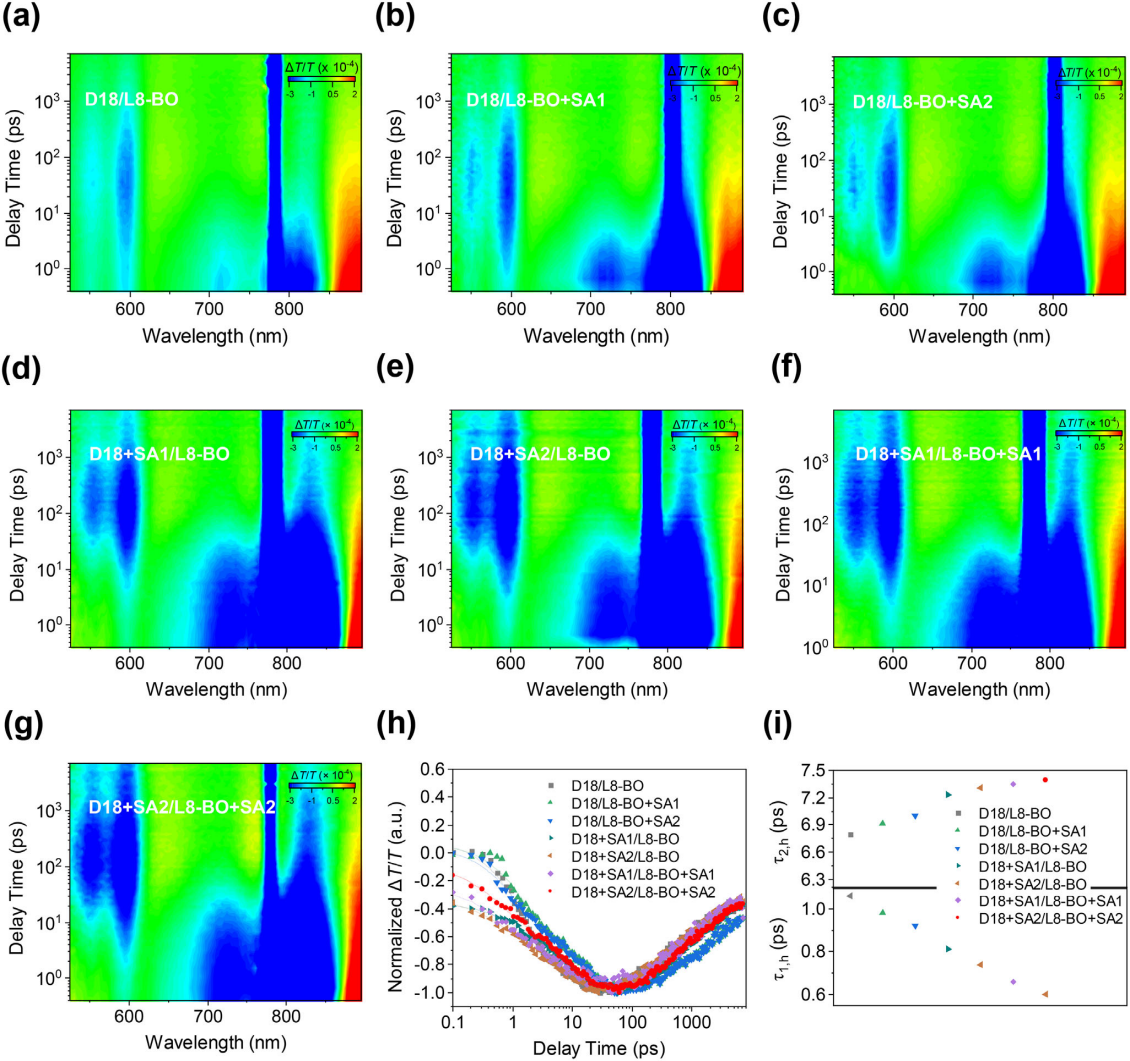
a–g) Femtosecond transient absorption spectroscopy spectra of top‐performing blend films. h) Decay dynamics of top‐performing blend films probed at 600 nm. i) Summary and comparison of *τ*_1,h_ and *τ*_2,h_ of the blend films.

The femtosecond transient absorption spectroscopy (fs‐TAS) techniques were conducted to estimate the exciton generation process to further investigate the improvement of photovoltaic performance by adding multifunctional solid additives. Using an excitation wavelength of 780 nm to excite the blend films to study the hole transport from the acceptor to the donor (**Figure** **3**a–g). The low energy excitation wavelength ensured that only the acceptor was excited but the donor was not affected. Ground‐state bleach (GSB) signal of the acceptor was observed ≈820 nm, in addition, the excited state absorption (ESA) band was also detected at 890 nm. The GSB signals of D18 were observed at 550 and 600 nm, which raised accompanied by the decay of the acceptor, indicating the hole transfer from the acceptor to the donor. Under the same conditions, from Figure 3a–g, the GSB signals of D18 increased gradually, suggesting more effective hole transfer. The hole transfer kinetics were discussed by fitting the GSB signals of D18 at 600 nm with a double‐exponential function (Figure 3h). The extracted time constants (*τ*_1,h_/*τ*_2,h_) were 1.06/6.80, 0.98/6.92, 0.92/7.00, 0.81/7.23, 0.74/7.31, 0.66/7.35, and 0.60/7.39 ps for D18/L8‐BO, D18/L8‐BO+SA1, D18/L8‐BO+SA2, D18+SA1/L8‐BO, D18+SA2/L8‐BO, D18+SA1/L8‐BO+SA1 and D18+SA2/L8‐BO+SA2 blend films (Figure 3i), respectively, where *τ*_1,h_ and *τ*_2,h_ represented the ultrafast dissociation of excitons at the donor/acceptor interfaces, and the time for excitons diffusing to the interfaces before dissociation,^[^ ^63^ ^]^ respectively. Clearly, the addition of multifunctional solid additives promoted more effective exciton dissociation and achieved more ideal donor/acceptor interfaces.

Analyzing the dependence of photocurrent (*J*_ph_) on effective voltage (*V*_eff_) to explore the effect of multifunctional solid additives on the exciton dissociation behaviors. Here, *J*_ph_ is the difference between *J*_light_ and *J*_dark_, where *J*_light_ and *J*_dark_ represent the photocurrent density under illumination and in the dark, respectively. Similarly, *V*_eff_ is the difference between *V*_0_ and *V*_a_, where *V*_0_ and *V*_a_ represent the voltage when *J*_ph_ is zero and the applied voltage, respectively. The exciton dissociation probability (*P*_(E, T)_) can be calculated from *J*_ph_/*J*_sat_ (where *J*_sat_ represents the saturated current density). Evidently, when *V*_eff_ reached 2 V, *J*_ph_ reached saturation, and all the devices showed very similar *J*_sat_ (Figure S30a, Supporting Information). The *P*_(E, T)_ was determined to be 97.10% for D18/L8‐BO based devices, while the devices based on D18/L8‐BO+SA1, D18/L8‐BO+SA2, D18+SA1/L8‐BO, D18+SA2/L8‐BO, D18+SA1/L8‐BO+SA1 and D18+SA2/L8‐BO+SA2 attained higher *P*_(E, T)_ of 97.43%, 97.50%, 97.76%, 97.90%, 98.39% and 98.60%, respectively, implying the application of multifunctional solid additives could enhance more efficient exciton dissociation to achieve higher *J*_SC_ and FF.

The charge transport properties were characterized by utilizing the space charge limited conductivity (SCLC) methods (Figure S31a,b, Supporting Information). The hole mobility (*µ*_h_) and electron mobility (*µ*_e_) of D18/L8‐BO, D18/L8‐BO+SA1, D18/L8‐BO+SA2, D18+SA1/L8‐BO, D18+SA2/L8‐BO, D18+SA1/L8‐BO+SA1 and D18+SA2/L8‐BO+SA2 based devices were estimated to be 5.31 × 10^−4^/4.79 × 10^−4^, 5.49 × 10^−4^/5.00 × 10^−4^, 5.55 × 10^−4^/5.09 × 10^−4^, 5.71 × 10^−4^/5.32 × 10^−4^, 5.88 × 10^−4^/5.55 × 10^−4^, 6.01 × 10^−4^/5.82 × 10^−4^ and 6.27 × 10^−4^/6.13 × 10^−4^ cm^2^ V^−1^ s^−1^, with the *µ*_h_/*µ*_e_ values of 1.11, 1.10, 1.09, 1.07, 1.06, 1.03, and 1.02, respectively. In addition, the photogenerated charge extraction by linearly increasing voltage (photo‐CELIV) tests was also carried out to further analyze the charge transport properties of the free charge carriers (Figure S31c, Supporting Information). According to the equation,^[^ ^64^ ^]^ the charge carrier mobility *µ*_celiv_ was determined to be 3.62 × 10^−4^, 3.70 × 10^−4^, 3.75 × 10^−4^, 3.87 × 10^−4^, 3.92 × 10^−4^, 4.28 × 10^−4^ and 4.44 × 10^−4^ cm^2^ V^−1^ s^−1^ for D18/L8‐BO, D18/L8‐BO+SA1, D18/L8‐BO+SA2, D18+SA1/L8‐BO, D18+SA2/L8‐BO, D18+SA1/L8‐BO+SA1 and D18+SA2/L8‐BO+SA2 based devices, respectively. Obviously, the devices containing multifunctional solid additives displayed higher charge mobility, especially the D18+SA1/L8‐BO+SA1 and D18+SA2/L8‐BO+SA2 based devices showed the most balanced charge mobility, which was also attributed to the synergistic effect of the multifunctional solid additives. The higher and more balanced charge mobility could suppress the charge accumulation and recombination as well as non‐radiative recombination energy loss.

The dependences of *J*_sc_ and *V*_oc_ on light intensity (*P*_light_) were conducted to understand the charge recombination mechanism (Figure S30b,c, Supporting Information).^[^ ^65^, ^66^, ^67^, ^68^, ^69^, ^70^ ^]^ The relationship of *J*_sc_ and *P*_light_ could be defined as *J*_sc_ ∝ *P*_light_ *^α^*, where *α* reflects the extent of biomolecular charge recombination. The extracted *α* values were 0.975, 0.977, 0.981, 0.984, 0.989 and 0.992 for D18 /L8‐BO+SA1, D18/L8‐BO+SA2, D18+SA1/L8‐BO, D18+SA2/L8‐BO, D18+SA1/L8‐BO+SA1 and D18+SA2/L8‐BO+SA2, which were higher than that of D18/L8‐BO (0.971). Furthermore, trap‐assisted recombination was studied by fitting the curve of the *V*_oc_ against *P*_light_. The slopes were found at 1.19 *kT*/*q*, 1.18 *kT*/*q*, 1.16 *kT*/*q*, 1.12 *kT*/*q*, 1.11 *kT*/*q*, 1.08 *kT*/*q* and 1.07 *kT*/*q* for D18/L8‐BO, D18/L8‐BO+SA1, D18/L8‐BO+SA2, D18+SA1/L8‐BO, D18+SA2/L8‐BO, D18+SA1/L8‐BO+SA1 and D18+SA2/L8‐BO+SA2 based devices, respectively. Apparently, the use of multifunctional solid additives could effectively suppress bimolecular recombination and reduce trap recombination. The suppressed bimolecular recombination and lower trap‐assisted recombination were beneficial for achieving higher *J*_sc_ and FF. The transient photovoltage (TPV) and transient photocurrent (TPC) measurements were used to probe the charge carrier lifetime (Figure S32a,b, Supporting Information). As expected, D18/L8‐BO+SA1, D18/L8‐BO+SA2, D18+SA1/L8‐BO, D18+SA2/L8‐BO, D18+SA1/L8‐BO+SA1 and D18+SA2/L8‐BO+SA2 based devices achieved longer charge carrier lifetime and shorter charge carrier extraction time of 4.48/0.48, 4.55/0.46, 4.67/0.43, 4.72/0.40, 5.10/0.37, and 5.18/0.35 µs than that of D18/L8‐BO (4.36/0.52 µs) based device, illustrating lower charge recombination rate and fast charge extraction could be reached by adding multifunctional solid additives. Compared with other devices, D18+SA1/L8‐BO+SA1 and D18+SA2/L8‐BO+SA2 devices presented excellent charge carrier dynamics, which was the result of the synergistic effect of the multifunctional solid additives.

**Figure 4:**
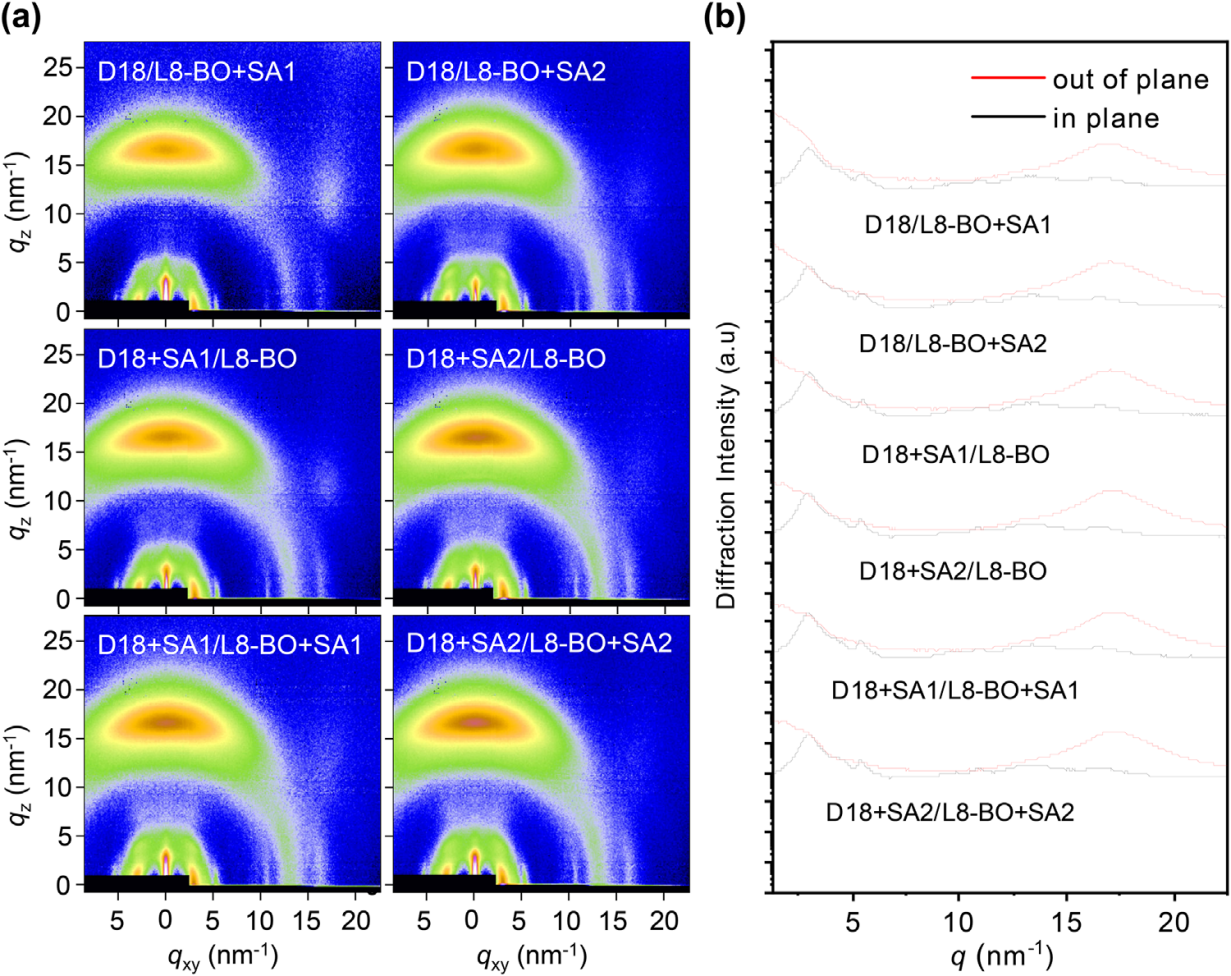
a) GIWAXS images of blend films. b) The corresponding out‐of‐plane line cuts (red lines) and in‐plane (black lines) and from (a).

The GIWAXS measurements were conducted to further understand the effect of multifunctional solid additives on the quality of blend films (**Figure** **4**a,b). All blend films remained face‐on orientation, contributing to the OOP charge transport. The D18/L8‐BO, D18/L8‐BO+SA1, and D18/L8‐BO+SA2 blend films demonstrated an identical CCL value of 9.82 nm for the (11$\bar{1}$) scatterings along the IP direction (Table S4, Supporting Information). For the D18+SA1/L8‐BO and D18+SA1/L8‐BO+SA1 blend films, as well as D18+SA2/L8‐BO and D18+SA2/L8‐BO+SA2 blend films, the corresponding CCL values increased to 10.36 and 10.56 nm, respectively. Besides, D18+SA1/L8‐BO and D18+SA1/L8‐BO+SA1 blend films, as well as D18+SA2/L8‐BO and D18+SA2/L8‐BO+SA2 blend films displayed significantly enhanced diffraction intensity in the OOP direction. These results could be attributed to the multifunctional solid additives enhancing the crystallinity of D18, which agreed well with the SCLC results.

**Figure 5:**
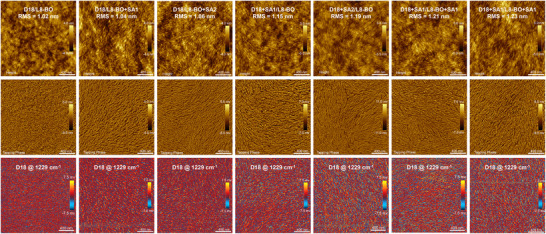
AFM images and PiFM images of the blend films. Top: surface height images; middle: tapping phase images; bottom: PiFM images.

Atomic force microscopy (AFM) and photo‐induced force microscopy (PiFM) measurements were further used to characterize the effect of multifunctional solid additives on the morphologies of the blended films (**Figure** **5**). As shown in AFM images, smooth morphologies were observed in the D18/L8‐BO, D18/L8‐BO+SA1, and D18/L8‐BO+SA2 blend films with smaller mean‐square of surface roughness (RMS) of 1.02, 1.04, and 1.06 nm, respectively. Furthermore, well‐defined nanofibers could be observed in the tapping phase images of D18/L8‐BO+SA1 and D18/L8‐BO+SA2 films. Due to the enhanced crystallinity, D18+SA1/L8‐BO, D18+SA2/L8‐BO, D18+SA1/L8‐BO+SA1, and D18+SA2/L8‐BO+SA2 blend films displayed nanofibers with slightly increased widths and lengths with the RMS raised to 1.15, 1.19, 1.21, and 1.23 nm, respectively. Especially, the D18+SA1/L8‐BO+SA1 and D18+SA2/L8‐BO+SA2 blend films exhibited appropriate nanofibers and the optimum phase separation with bicontinuous network, attributed from the synergistic effect of the multifunctional solid additives. More importantly, the dispersion improved gradually from D18/L8‐BO to D18/L8‐BO+SA1, D18/L8‐BO+SA2, D18+SA1/L8‐BO, D18+SA2/L8‐BO, D18+SA1/L8‐BO+SA1, and D18+SA2/L8‐BO+SA2 blend films, suggesting the multifunctional solid additives could promote the formation of more ideal interpenetrating network morphology, which was conducive to exciton diffusion and charge transport, thus enabling lower *E*_loss_ and higher PCE.

## 3 Conclusion

In this work, we successfully designed and synthesized two multifunctional solid additives of SA1 and SA2, by just replacing BT in Y6 skeleton with NT and incorporating different terminal groups. If these multifunctional solid additives were added to D18 layer, it could enhance the crystallinity and ordered molecular stacking of D18, thus reducing the related energy disorder (Δ*E*_2_). On the other hand, if incorporating multifunctional solid additive into L8‐BO layer, it just optimized the morphology, leading to a reduction in Δ*E*_3_. Notably, the simultaneously adding multifunctional solid additive into D18 and L8‐BO layers would exhibit a significant synergistic effect, finally leading to a remarkable improvement in overall device performance with the highest PCE of 19.95% and the lowest *E*_loss_ of 0.532 eV, where Δ*E*_2_ and Δ*E*_3_ decreased to 0.055 and 0.199 eV, respectively. This additive simultaneous incorporation into the donor and acceptor layers strategy demonstrated the potential for significantly enhancing the photovoltaic performance of LBL PSCs by reducing energy disorder and optimizing morphology.

## Conflict of Interest

The authors declare no conflict of interest.

## Supporting information



Supporting Information

## Data Availability

Research data are not shared.
