# Supplementary material for: Minimizing Energy Loss by Designing Multifunctional Solid Additives to Independent Regulation of Donor and Acceptor Layers for Efficient LBL Polymer Solar Cells
Source: Adv Sci (Weinh). 2025 Mar 20;12(18):2414712. doi: 10.1002/advs.202414712 (PMC12079416; doi:10.1002/advs.202414712)
Supplement: Supplementary file 1 — Supporting Information [file ADVS-12-2414712-s001.docx]

**Supporting Information**

**Minimizing Energy Loss by Designing Multifunctional Solid Additives to Independent Regulation of Donor and Acceptor Layers for Efficient LBL Polymer Solar Cells**

*Junying Wang, Min Deng,* Haonan Chen, Wuke Qiu, Yuwei Duan, Chentong Liao, Ruipeng Li, Liyang Yu, Qiang Peng**

J. Y. Wang, Dr. M. Deng, H. N. Chen, Dr. Y. W. Duan, Dr. C. T. Liao, Prof. Q. Peng

College of Materials and Chemistry & Chemical Engineering, Chengdu University of Technology, Chengdu 610059, P. R. China

E-mail: [mindeng@cdut.edu.cn](mailto:mindeng@cdut.edu.cn); [qiangpeng@scu.edu.cn](mailto:qiangpeng@scu.edu.cn)

Dr. W. K. Qiu, Dr. L. Y. Yu, Prof. Q. Peng

School of Chemical Engineering and State Key Laboratory of Polymer Materials Engineering, Sichuan University, Chengdu 610065, P. R. China

E-mail: [qiangpeng@scu.edu.cn](mailto:qiangpeng@scu.edu.cn)

Dr. R. P. Li

National Synchrotron Light Source II, Brookhaven National Lab, Suffolk, Upton, NY 11973, USA

**1. Materials and Methods**

**Materials:** D18 and L8-BO was purchased from Solarmer Material Inc. 2-(5, 6-difluoro-3-oxo-2,3-dihydro-1H-inden-1-ylidene)malononitrile (IC-2F) and 2-(5,6-dichloro-3-oxo-2,3-dihydro-1H-inden-1-ylidene)malononitrile (IC-2Cl) were purchased from Derthon Optoelectronics Materials Science Technology Co., LTD. 2PACZ was purchased from Tansoole. Compound 1 and compound 3 were synthesized according to previously reported procedures.^[1, 2]^ All the other chemicals were purchased from Aladdin, Adamas, Sigma-Aldrich and Alfa Asear Chemical Co., and used without further purification. All solvents were freshly distilled immediately prior to use.

**Synthesis of compound 2:** In a round bottom flask, compound 1 (1.00 g, 2.50 mmol) was added into fuming nitric acid (5.0 mL) at 0 ^o^C. Then, trifluoromethanesulfonic acid (15.0 mL) was added dropwise and the mixture was stirred at 60 ^o^C for 24 h. After cooling to room temperature, the mixture was poured into ice water slowly, and the precipitate was collected by filtration. Recrystallization in ethanol yielded compound 2 as a tan solid (0.16 g, 13.28%). MS: m/z calcd for C_10_Br_2_N_6_O_4_S_2_: 491.7769, found: 492.7797.

**Synthesis of compound 4:** In a round bottom flask, compound 2 (0.50 g, 1.02 mmol), compound 3 (1.79 g, 3.06 mmol) and Pd(PPh_3_)_4_ (0.060 g, 0.051 mmol) were dissolved in 1,2-dichlorobenzene (30.00 mL), then the mixture was refluxed under an argon atmosphere for 15 h. After cooling to room temperature, the solvent was concentrated by evaporation under reduced pressure and the residue was purified by chromatography with petroleum ether (PE)/ dichloromethane (DCM) (2:3, v/v) as the eluent to give compound 4 as a red solid (0.26 g, 28.31%). ^1^H NMR (400 MHz, CDCl_3_, δ/ppm) 7.843 (s, 2H), 7.185 (s, 2H), 2.816-2.778 (t, *J* = 15.20 Hz, 4H), 1.842-1.767 (m, 4H), 1.433-1.265 (m, 32H), 0.887-0.853 (t, *J* = 13.60 Hz, 6H). ^13^C NMR (100 MHz, CDCl_3_, δ/ppm) 152.795, 147.838, 145.361, 144.252, 139.015, 135.099, 130.131, 124.982, 124.332, 122.437, 116.651, 31.916, 29.881, 29.663, 29.634, 29.586, 29.412, 29.394, 29.349, 28.601, 22.689, 14.123. MALDI-TOF: m/z calcd for C_44_H_50_N_6_O_4_S_6_: 919.225, found: 919.295.

**Synthesis of compound 5:** In a round bottom flask, compound 4 (1.30 g, 1.41 mmol), triethyl phosphite (28.0 mL) and 1,2-dichlorobenzene (12.0 mL) were mixed under N_2_ atmosphere. Then, the mixture was heated to 180 ^o^C for 24 h. After cooling to room temperature, the solvent was concentrated by evaporation under reduced pressure. Without further purification, potassium iodide (2.34 g, 14.10 mmol), potassium carbonate (1.95 g, 14.10 mmol), 5-(bromomethyl)undecane (3.51 g, 14.10 mmol) and anhydrous *N,N*-dimethylformamide (18.0 mL) were added into the residue. The mixture was stirred at 120 ^o^C for 24 h. After cooling to room temperature, the mixture was poured into water and extracted with dichloromethane. The organic layer was dried over anhydrous MgSO_4_ and concentrated by evaporation under reduced pressure. Compound 5 was very unstable and the yield was also extremely low. So, without any further purification, compound 5 was used into the following reaction. MALDI-TOF: m/z calcd for C_68_H_98_N_6_S_6_: 1191.621, found: 1191.623.

**Synthesis of compound SA-CHO:** In a two neck round bottomed flask, anhydrous *N,N*-dimethylformamide (2.0 mL) and 1,2-dichloroethane (10.0 mL) were added and cooled to 0 ^o^C under an argon atmosphere. Then, phosphorous oxychloride (2.0 mL) was added slowly and stirred at this temperature for 2 h, and then compound 5 (0.10 g, 0.084 mmol) was added. When finishing this adding, the mixture was stirred at 110 ^o^C for 12 h. After cooling to room temperature, the mixture was poured into water and extracted with dichloromethane. The organic layer was dried over anhydrous MgSO_4_ and concentrated by evaporation under reduced pressure. The pure product of compound 6 was obtained by column chromatography with PE/DCM (2:3, v/v) as the eluent to give a light red solid. (0.040g, 38.26%). ^1^H NMR (400 MHz, CDCl_3_, δ/ppm) 10.159 (s, 2H), 3.236-3.198 (t, *J* = 15.20 Hz, 4H), 1.973-1.898 (m, 4H), 1.488-1.450 (t, *J* = 15.20 Hz, 4H), 1.320-1.195 (m, 34H), 0.876-0.675 (m, 44H), 0.519-0.485 (t, *J* = 13.60 Hz, 6H). ^13^C NMR (100 MHz, CDCl_3_, δ/ppm) 181.918, 149.327, 148.194, 146.622, 143.721, 139.725, 137.912, 137.423, 129.505, 124.146, 117.179, 112.988, 55.951, 38.645, 31.916, 31.414, 30.740, 30.479, 30.345, 29.707, 29.651, 29.615, 29.535, 29.398, 29.339, 29.215, 28.111, 27.909, 25.756, 25.731, 22.694, 22.547, 22.403, 14.128, 13.992, 13.596. MALDI-TOF: m/z calcd for C_70_H_98_N_6_O_2_S_6_: 1247.611, found: 1247.610.

**Synthesis of compound SA1:** In a round bottom flask, compound 6 (0.10 g, 0.080 mmol), 2-(5,6-difluoro-3-oxo-2,3-dihydro-1H-inden-1-ylidene)malononitrile (0.18 g, 0.80 mmol) were dissolved in chloroform (35.0 mL) and then added pyridine (1.5 mL). The mixture was heated to 90 ^o^C for 15 h. The mixture was directly purified by silica gel column chromatography by using chloroform as the eluent to yield the compound SA1 as a black solid. (0.10 g, 75.93%). ^1^H NMR (400 MHz, CDCl_3_, δ/ppm) 8.766 (s, 2H), 8.437-8.398 (t, *J* = 15.60 Hz, 2H), 7.550-7.516 (t, *J* = 13.60 Hz, 2H), 3.081 (s, 4H), 1.813-1.794 (t, *J* = 7.60 Hz, 4H), 1.479-1.367 (m, 4H), 1.335-1.257 (m, 34H), 0.953-0.684 (m, 44H), 0.501 (s, 6H). ^13^C NMR (100 MHz, CDCl_3_, δ/ppm) 185.356, 175.420, 158.467, 155.442, 153.225, 149.285, 148.399, 145.153, 139.911, 139.616, 136.319, 135.189, 134.460, 133.106, 129.912, 127.100, 119.844, 117.761, 114.984, 114.567, 114.428, 113.317, 68.706, 56.416, 39.022, 31.975, 31.489, 31.141, 30.829, 29.788, 29.666, 29.527, 29.458, 29.371, 27.270, 22.740, 22.618, 22.445, 14.165, 14.060, 13.644. MALDI-TOF: m/z calcd for C_94_H_102_F_4_N_10_O_2_S_6_: 1671.648, found: 1671.660.

**Synthesis of compound SA2:** In a round bottom flask, compound 6 (0.10 g, 0.080 mmol), 2-(5,6-dichloro-3-oxo-2,3-dihydro-1H-inden-1-ylidene)malononitrile (0.21 g, 0.80 mmol) were dissolved in chloroform (35.0 mL) and then added pyridine (1.5 mL). The mixture was heated to 92 ^o^C for 18 h. The mixture was directly purified by silica gel column chromatography by using chloroform as the eluent to yield the compound SA2 as a black solid. (0.12 g, 82.77%). ^1^H NMR (400 MHz, CDCl_3_, δ/ppm) 8.785 (s, 2H), 8.599 (s, 2H), 7.850 (s, 2H), 3.025 (s, 4H), 1.766-1.692 (m, 4H), 1.438-1.402 (t, *J* = 14.40 Hz, 4H), 1.339-1.264 (t, *J* = 30.00 Hz, 34H), 0.975-0.665 (m, 44H), 0.476 (s, 6H). ^13^C NMR (100 MHz, CDCl_3_, δ/ppm) 187.932, 186.458, 150.920, 149.345, 148.525, 144.721, 140.036, 139.436, 139.145, 138.968, 138.933, 138.819, 133.638, 133.010, 132.796, 126.119, 124.134, 120.648, 117.724, 112.975, 38.900, 31.927, 31.482, 30.681, 29.819, 29.668, 29.639, 29.560, 29.464, 29.351, 29.308, 22.705, 22.583, 22.431, 14.135, 14.031, 13.637. MALDI-TOF: m/z calcd for C_94_H_102_Cl_4_N_10_O_2_S_6_: 1737.527, found: 1737.590.

**2. Material characterizations**

^1^H and ^13^C NMR spectra were recorded on a Bruker Avance-400 spectrometer with *d*-chloroform as solvent. The chemical shifts were reported as δ value (ppm) relative to an internal tetramethylsilane (TMS) standard. UV-vis spectra were obtained on a Hitachi U2910 spectrophotometer. Femtosecond transient absorption spectroscopy (fs-TAS) was conducted using a commercial Helios setup from Ultrafast Systems with a Ti:sapphire regenerative amplified laser system (Coherent Libra) delivered laser pulses at 780 nm (100 fs, 1 kHz). An optical parametric amplifier (Vitara, Coherent) pumped by the regenerative amplifier was used to generate the pump beam at 600 nm (at wavelength resonant with the absorption of D18). The probe beam was generated by focusing part of the fundamental femtosecond laser beam onto a sapphire plate or Yttrium aluminum garnet plate for visible (vis) and near-IR (NIR) spectral windows, respectively. TA results in this work are presented in the unit of ΔOD, negative features can reflect ground-state bleaching (GSB) or stimulated emission (SE), a positive signal is an excited-state absorption (ESA). During TA measurements, the samples were kept in nitrogen to avoid photodegradation. The pump fluence was kept at <5 μJ cm^-2^ to minimize the exciton-exciton annihilation effect. GIWAXS measurements were performed at Complex Materials Scattering (CMS) beamline of the National Synchrotron Light Source II (NSLS-II), Brookhaven National Lab. AFM images were obtained by using a Bruker Nano IR-3 atomic microscope in tapping mode. The PiFM images were collected on a Bruker NanoIR-3 atomic force microscope in contact mode.

**Urbach energy (*E*_U_) Calculation**

The *E*_U_ was measured by fitting the tail state of the EQE curves,^[3, 4]^ and the relationship of absorption coefficient (*α*) and EQE can be defined as:

$\text{α}\text{ = -}\frac{\text{ln(1-EQE)}}{\text{d}}$ (1)

In the low absorption region (*αd* ≤ 1), it can be approximated as:

$\text{α}\text{ = }\frac{\text{EQE}}{\text{d}}$ (2)

Select the region with photon energy lower than *E*_g_, according to the equation:

*α*(E) ∝ $\text{e}^{{\text{E}\text{/}\text{E}}_{\text{U}}}$ (3)

By taking the logarithm on both sides:

$\text{ln}\text{α}\text{= }\frac{\text{E}}{\text{E}_{\text{U}}}\text{ + B}$ (4)

Where B is the constant.

Linear fitting is performed on ln*α* and *E*, and the fitted slope is 1/*E*_U_. The calculation formula for *E*_U_ is as follows:

*E*_U_ = 1/slope (5)

**Voltage Loss Calculation**

The *V*_oc_ of solar cells can be calculated in the following expression^[5]^:

$V_{\mathrm{oc}}=\frac{kT}{q}ln(\frac{J_{\mathrm{sc}}}{J_{0}}+1)$ (1)

Where *k* is the Boltzmann constant, *T* is the temperature, and *q* is the elementary charge.

The *J*_sc_ and *J*_0_ can be calculated by:

$J_{\mathrm{sc}}=q*\int_{0}^{\infty} {EQE}_{PV}\left( E \right)*\phi_{AM1.5}(E)dE$ (2)

$J_{0}=\frac{q}{{EQE}_{EL}}*\int_{0}^{\infty} {EQE}_{PV}\left( E \right)*(E)dE$ (3)

The expression for *J*_0_ is the Rau’s reciprocity relation,^[6]^ where *EQE_EL_* is radiative quantum efficiency of solar cells when charge carriers are injected into the devices in dark, and *ϕ*_BB_ is the black body spectrum.

When all the recombination is radiative, *J*_0_ is minimized, and *V*_oc_ is maximized:

$J_{0,rad}=q*\int_{0}^{\infty} {EQE}_{PV}\left( E \right)*\phi_{BB}(E)dE$ (4)

$V_{oc, rad}=\frac{kT}{q}\ln\left( \frac{J_{\mathrm{sc}}}{J_{0}}+1 \right)=\frac{kT}{q}\ln\left( \frac{q*\int_{0}^{\infty} {EQE}_{PV}\left( E \right)*(E)dE}{q*\int_{0}^{\infty} {EQE}_{PV}\left( E \right)*\phi_{BB}(E)dE}+1 \right)$ (5)

In the Shockley-Queisser theory,^[7]^ the general quantum efficiency *EQE*_PV, SQ_ can be defined as:

${EQE}_{PV,SQ}\left( E \right)=1, E\geq E_{g}; {EQE}_{PV,SQ}\left( E \right)=0, E<E_{g}$ (6)

Substituting general quantum efficiency *EQE*_PV, SQ_ (E) (equation 6) in equation 4, then the saturation current in SQ limit, *J*_0, SQ_ can be obtained:

$J_{0,SQ}=q*\int_{E_{g}}^{\infty} {EQE}_{PV,SQ}\left( E \right)*\phi_{BB}\left( E \right)dE=q*\int_{E_{g}}^{\infty} \phi_{BB}\left( E \right)dE$ (7)

In the same way, the value of the SQ open-circuit voltage limit, *V*_oc, SQ_ can be calculated according to equation 5:

$V_{oc,SQ}=\frac{kT}{q}\ln\left( \frac{J_{\mathrm{sc}}}{J_{0,SQ}}+1 \right)=\frac{kT}{q}\ln\left( \frac{q*\int_{0}^{\infty} {EQE}_{PV,SQ}\left( E \right)*(E)dE}{q*\int_{E_{g}}^{\infty} {EQE}_{PV,SQ}\left( E \right)*\phi_{BB}(E)dE}+1 \right)$ (8)

The difference between *V*_oc, SQ_ and *V*_oc, rad_ is due to that in the SQ theory, the band edge of the absorber is totally abrupt when calculating *V*_oc, SQ_. For *V*_oc, rad_, the band gap will be smeared out for the existence of charge transfer state absorption.

Therefore, we can deduce the voltage loss of radiative recombination, *V*_oc, rad_:

Δ*V*_oc, rad_ = *V*_oc, SQ_ - *V*_oc, rad_ (9)

The voltage loss due to non-radiative recombination (*V*_oc, non-rad_) can be rewritten as

Δ*V*_oc, non-rad_ = *V*_oc, rad_ - *V*_oc_ = -*q*^-1^*kT*(ln*EQE_EL_*) (10)

Based on the previous discussions, we are now able to summarize the energy loss from the *E*_g_ to the *qV*_oc_ for any type of solar cells:

*qV*_loss_ = Δ*V*_1_ + Δ*V*_2_ + Δ*V*_3_ = (*E*_g_ - *qV*_oc,SQ_) + *qV*_oc, rad_ + *qV*_oc, non-rad_ (11)

Therefore, each parameter in the energy loss section can be obtained based on related calculation.

**3. Fabrication and characterization of polymer solar cells**

**Fabrication of polymer solar cells devices**

The PSCs devices were fabricated in a conventional architecture of glass substrate/ITO/2PACz/donor/acceptor/PNDIT-F3N/Ag. The ITO glasses were treated with UV-ozone for 25 mins after being washed with soapy water, acetone and isopropanol alcohol, sequentially. The 2PACZ layer was fabricated by spin coating ethanol solution containing 2PACZ (1 mg mL^-1^) at a speed of 3000 rpm and then annealed at 80 ^o^C for 5 mins. The D18 (5 mg mL^-1^ in chloroform) solution or a mixed solution of D18 and 2% weight ratio of SA1 or SA2 was spin-coated on the substrates to form the donor layer with a thickness about 60 nm. The L8-BO (7.5 mg mL^-1^ in chloroform) solution or a mixed solution of L8-BO and 6% weight ratio of SA1 or SA2 was spin-coated on the top of the donor layer with a thickness approximately 40 nm. Then, the substrates were annealed at 80 ^o^C for 6 min. PNDIT-F3N was deposited on to the active layer by spin coating from a 0.5 mg mL^-1^ methanol solution. The silver electrode (~100 nm) was deposited by thermal evaporation in vacuum.

**Characterization of polymer solar cells**

Hole-only and electron-only devices for SCLC measurements were fabricated with similar methods in architectures of ITO/PEDOT:PSS/active layer/Au and ITO/ZnO/active layer/PNDIT-F3N/Ag, respectively. The ZnO layer was fabricated using a sol-gel method, and gold electrodes were deposited by thermal evaporation in vacuum. All device fabrications were performed in an argon gas filled glove box except for the deposition of PEDOT:PSS, 2PACZ and ZnO layers. The *J-V* characteristics of devices were recorded using a Keithley 2400 Source Measure Unit under AM1.5 G (100 mW cm^-2^) irradiation generated by a SAN-EI XES-70S1 solar simulator in an argon gas filled glove box. The EQE responses from PSCs devices were recorded on an Enlitech QE-R solar quantum efficiency testing system in air. PSCs devices for EQE, charge generation and charge recombination evaluations were the top performing devices. Charge carrier mobilities were tested using the space charge limited current (SCLC) method on hole-only and electron-only devices. The *J*-V curves measured on hole-only and electron-only devices were fitted to *J* = 9*ε_0_ε_r_μV*^2^/8*L*^3^, where *J* is the current density, *L* is the film thickness of the active layer, *μ* is the hole or electron mobility, *ε_r_* is the relative dielectric constant of the transport medium, *ε_0_* is the permittivity of free space (8.85×10^-12^ F m^-1^), *V* is the internal voltage in the device and *V* = *V*_appl_-*V*_bi_-*V*_a_, where *V*_appl_ is the applied voltage to the device, *V*_bi_ is the built-in voltage and *V*_a_ is the voltage drop. Transient photovoltage (TPV), transient photocurrent (TPC) and photogenerated charge extraction by linearly increasing voltage (photo-CELIV) mobilities data were obtained by the all-in-one characterization platform, Paios (Fluxim AG, Switzerland). In the TPV measurement, the settling time was 10 ms, pulse length was 10 µs and the follow-up time was 500 µs. In the TPC measurement, the settling time was 5 us, pulse length was 5 µs and the follow-up time was 50 µs. In the photo-CELIV measurement, the delay time was set to 0 us, the light intensity was 100%, the light-pulse length was 100 µs, finally the sweep ramp rate rose from 20 V ms^-1^ to 100 V ms^-1^. EQE_EL_ values were obtained from an in-house-built system including a Hamamatsu silicon photodiode 1010B, a Keithley 2400 SourceMeter to provide voltage and inject current, and a Keithley 6482 Picoammeter to measure the emitted light intensity. FTPS-EQE was measured using an integrated system (PECT-600, Enlitech), where the photocurrent was amplified and modulated by a lock-in instrument.

**4. Supporting Figures**

**Figure S1.** ^1^H NMR spectrum of compound 4.

**Figure S2.** ^13^C NMR spectrum of compound 4.

**Figure S3.** ^1^H NMR spectrum of compound SA-CHO.

**Figure S4.** ^13^C NMR spectrum of compound SA-CHO.

**Figure S5.** ^1^H NMR spectrum of compound SA1.

**Figure S6.** ^13^C NMR spectrum of compound SA1.

**Figure S7.** ^1^H NMR spectrum of compound SA2.

**Figure S8.** ^13^C NMR spectrum of compound SA2.


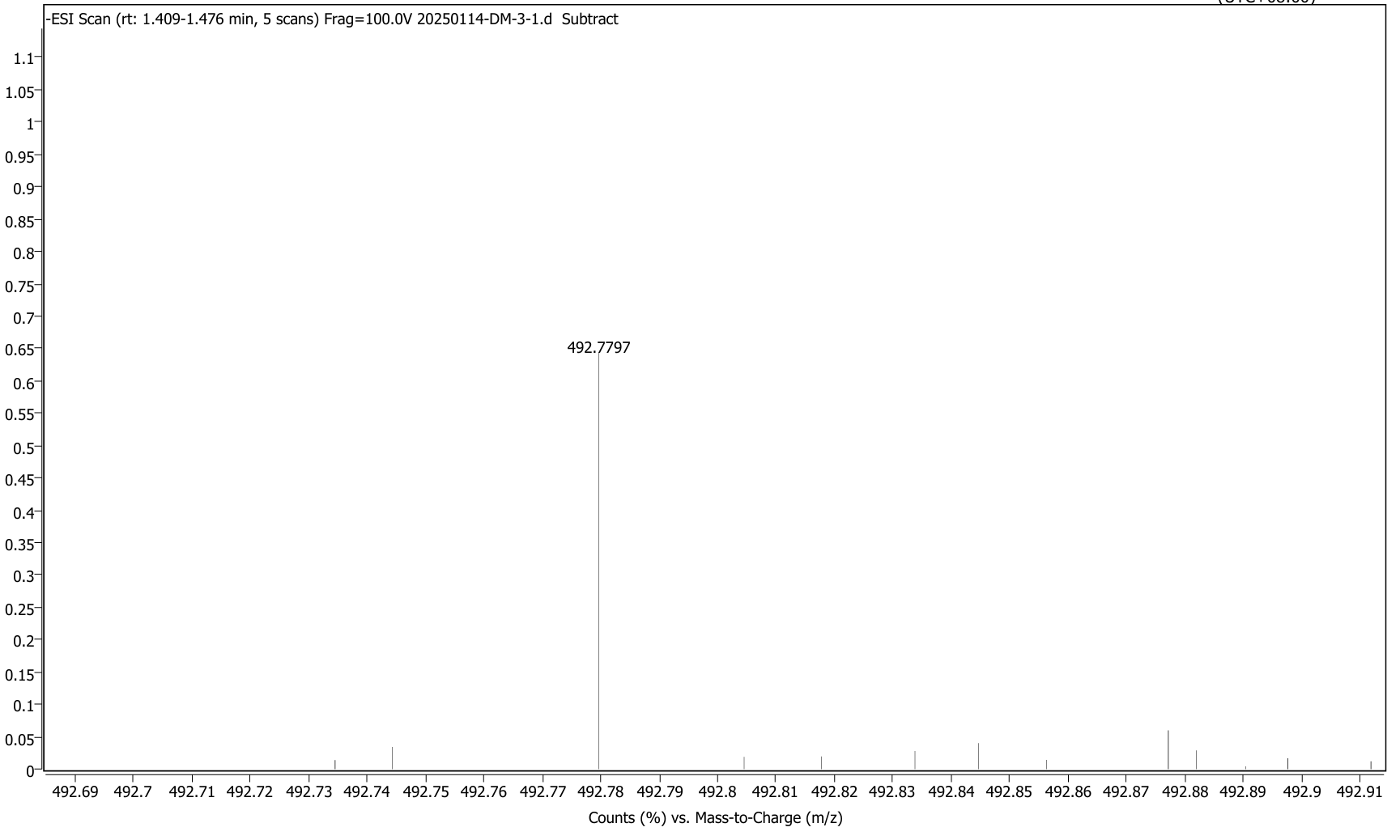


**Figure S9.** Mass spectrum of compound 2.


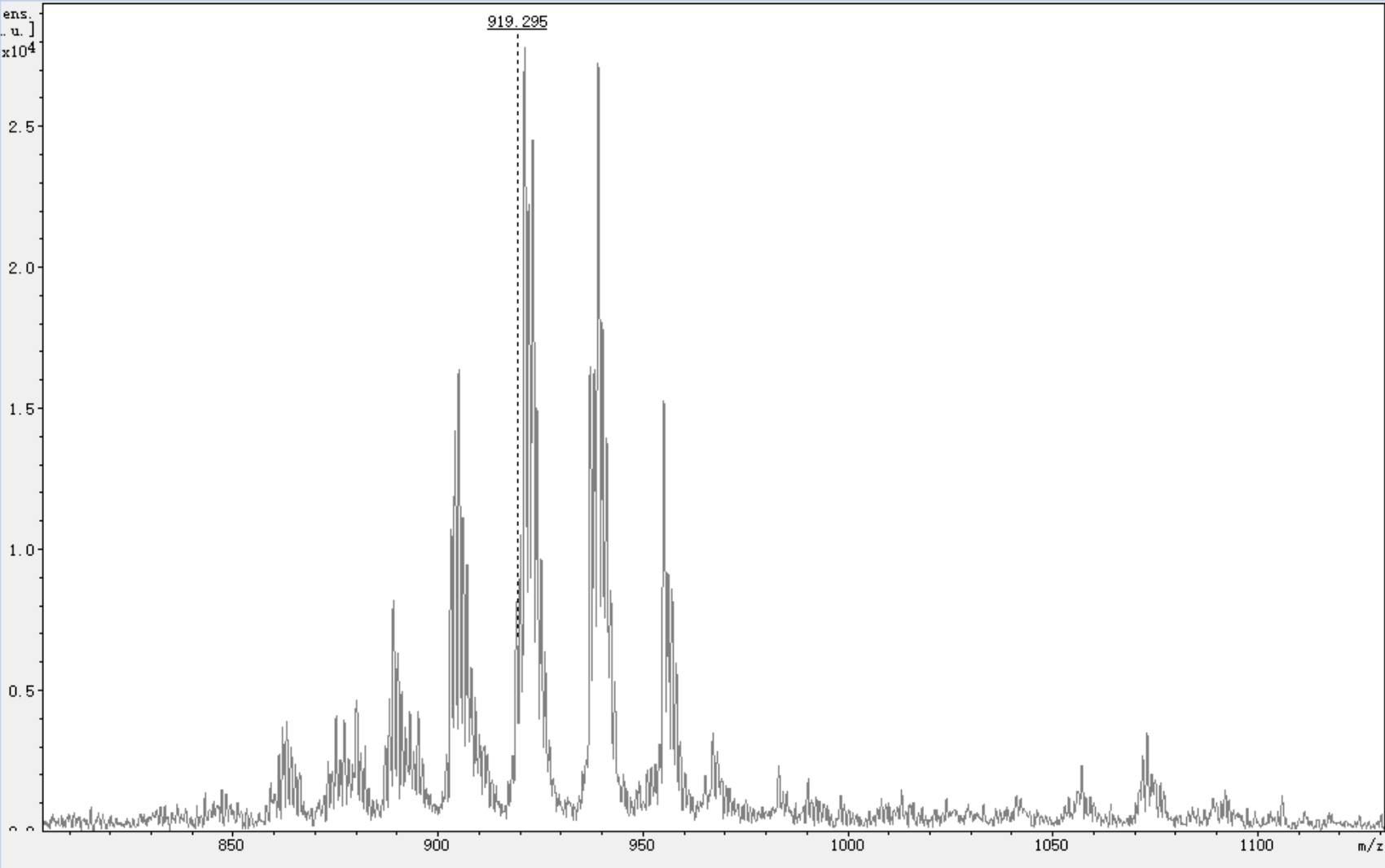


**Figure S10.** Mass spectrum (MALDI-TOF) of compound 4.


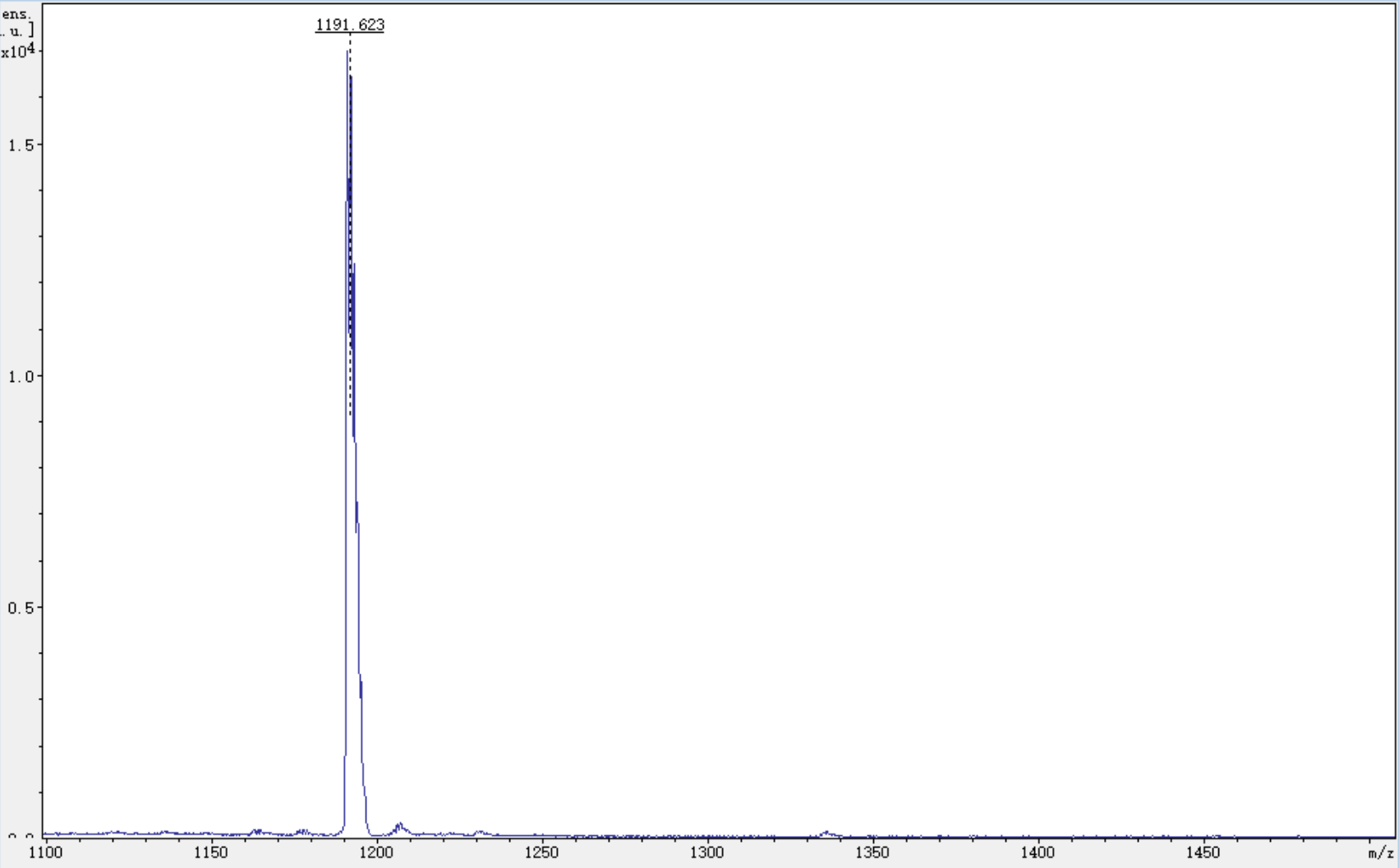


**Figure S11.** Mass spectrum (MALDI-TOF) of compound 5.


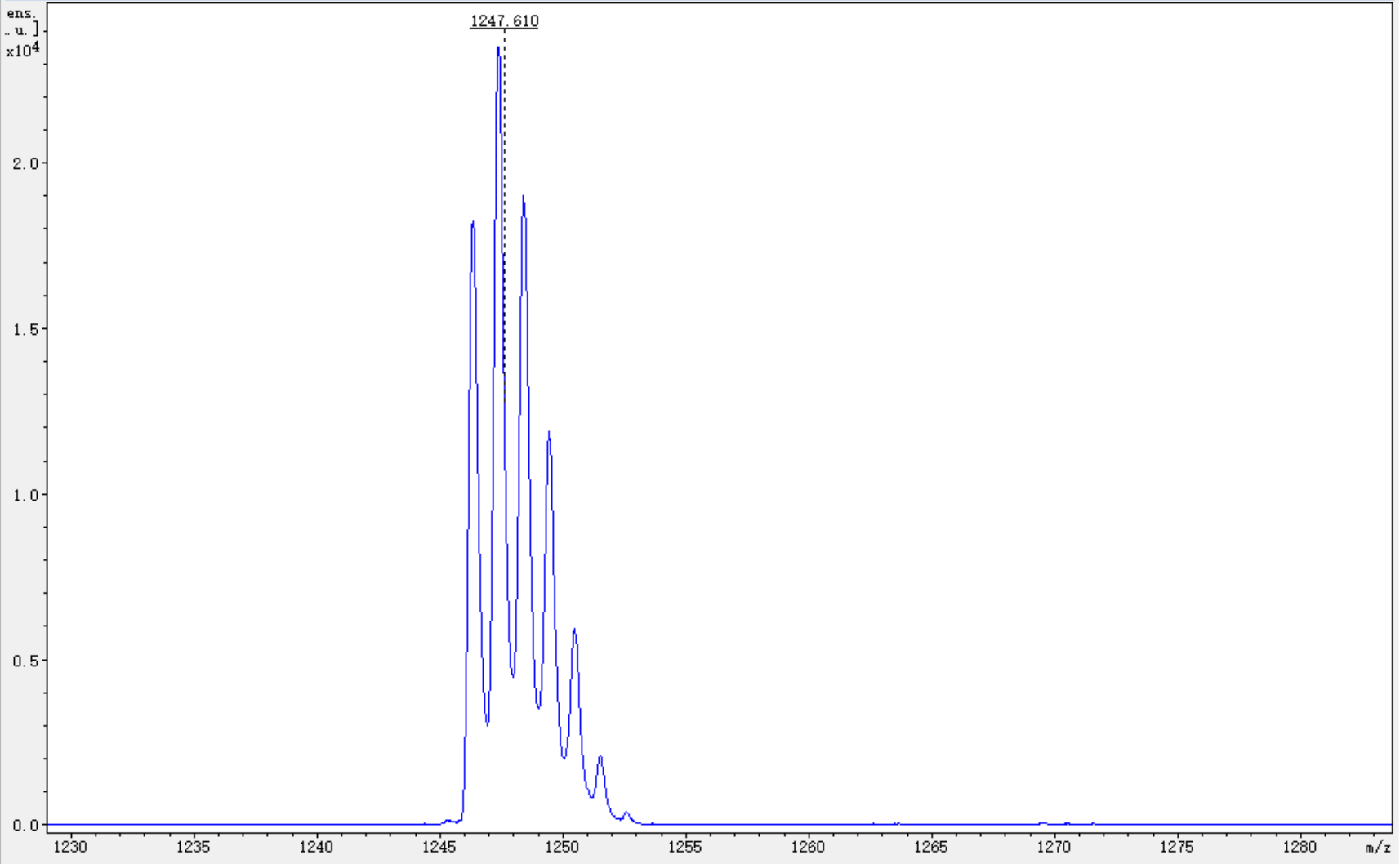


**Figure S12.** Mass spectrum (MALDI-TOF) of compound SA-CHO.


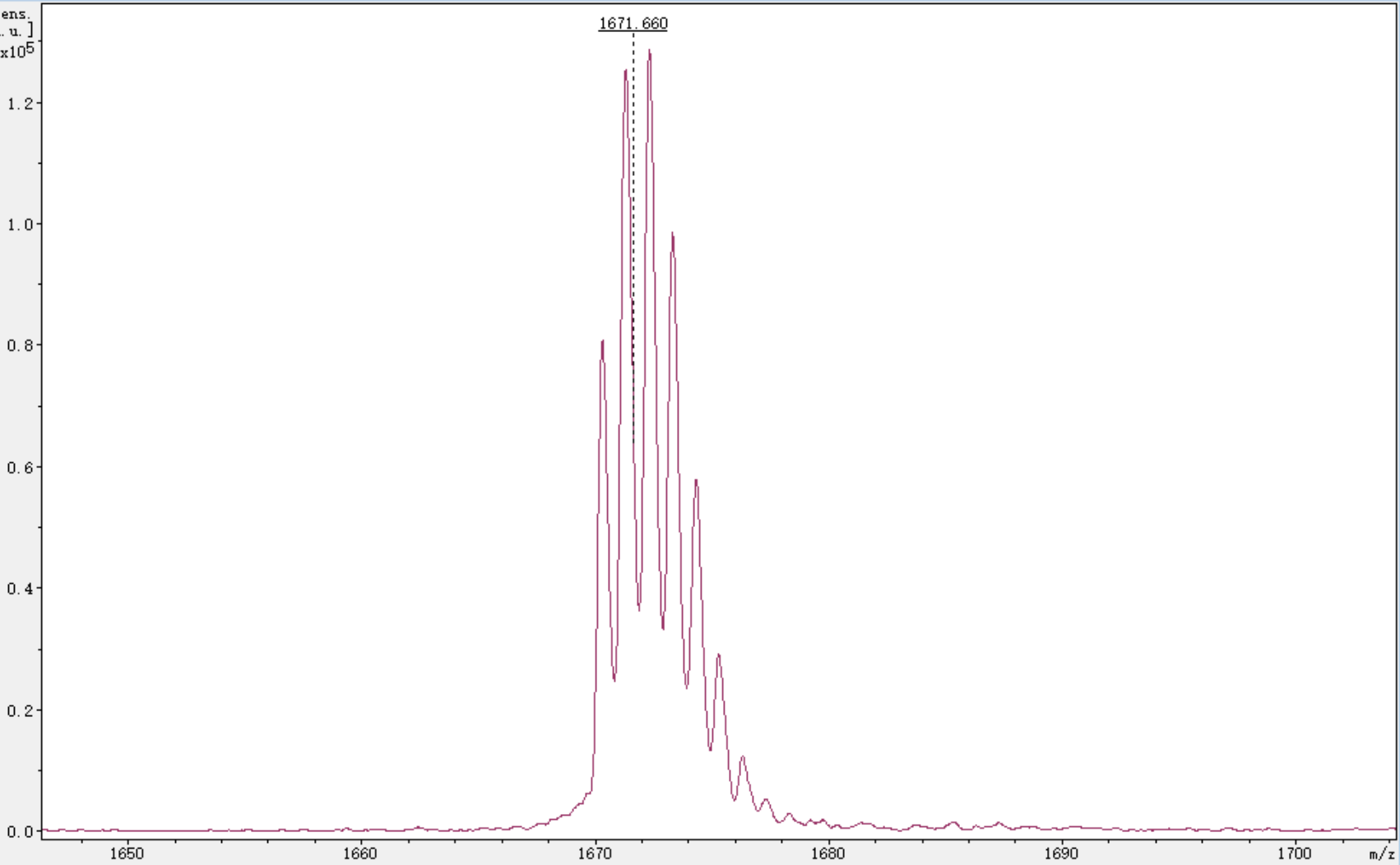
**Figure S13.** Mass spectrum (MALDI-TOF) of compound SA1.


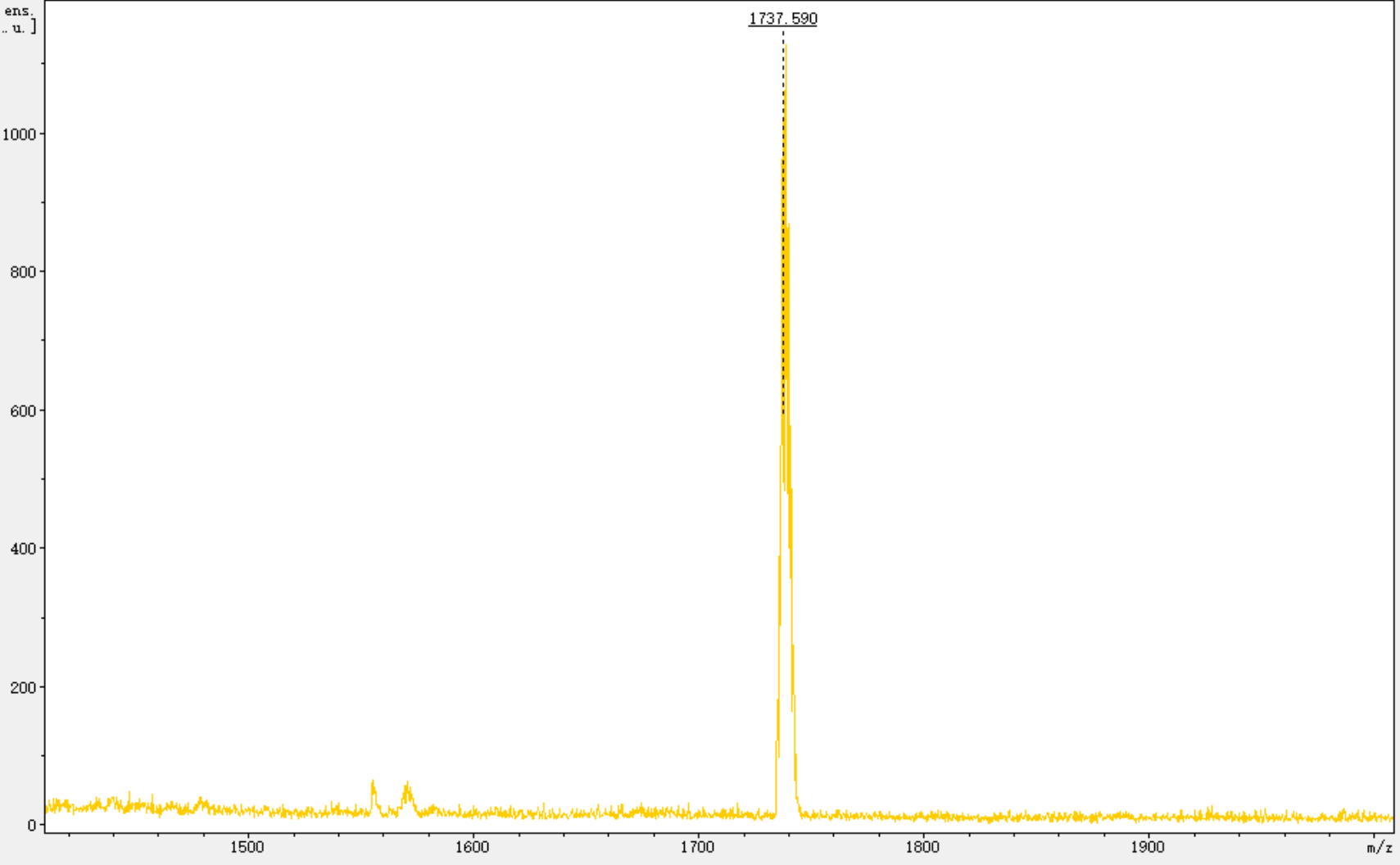


**Figure S14.** Mass spectrum (MALDI-TOF) of compound SA2.


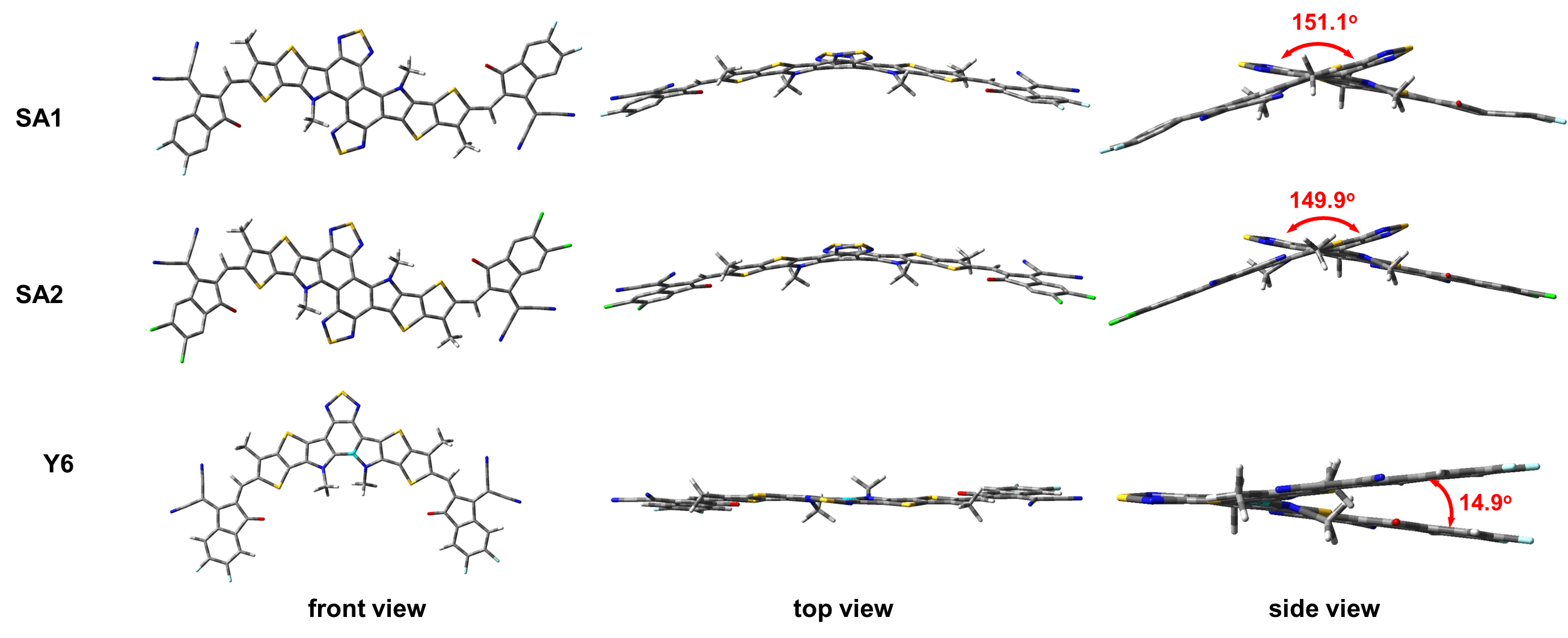


**Figure S15.** DFT calculation for geometry optimization for SA1, SA2 and Y6.


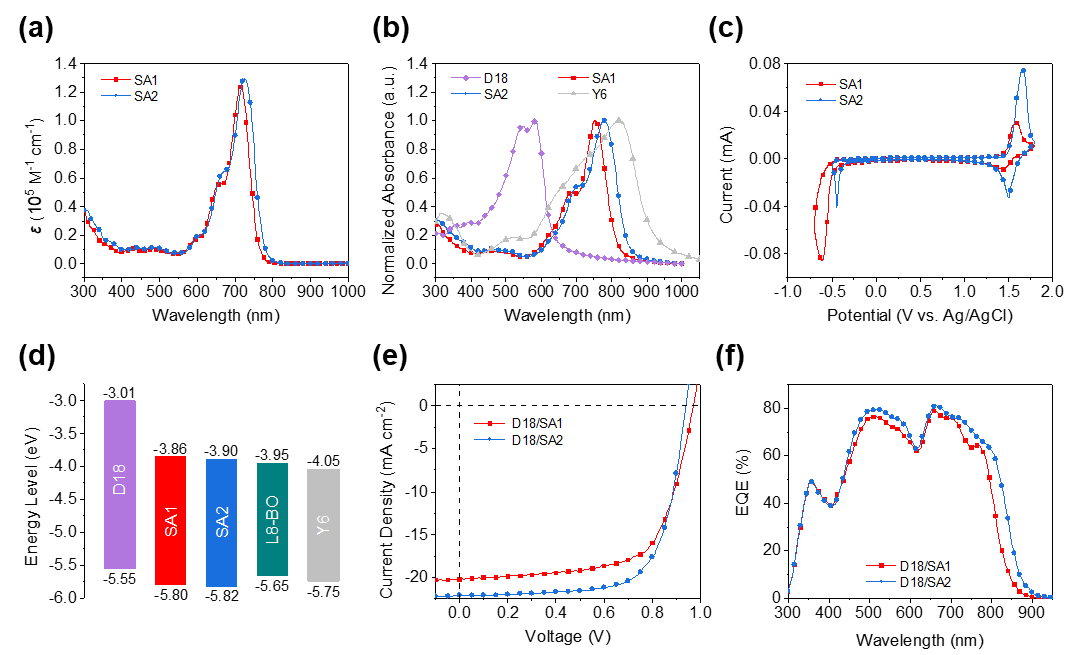


**Figure S16.** a) Molar extinction coefficient curves of SA1 and SA2 in dilute chloroform solution. b) Normalized absorption spectra of relative materials in solid-state thin films. c) CV curves of SA1 and SA2. d) Energy level diagram of relative materials. e) *J*-*V* curves and (f) EQE curves of the top-performing devices based on D18/SA1 and D18/SA2 blends.


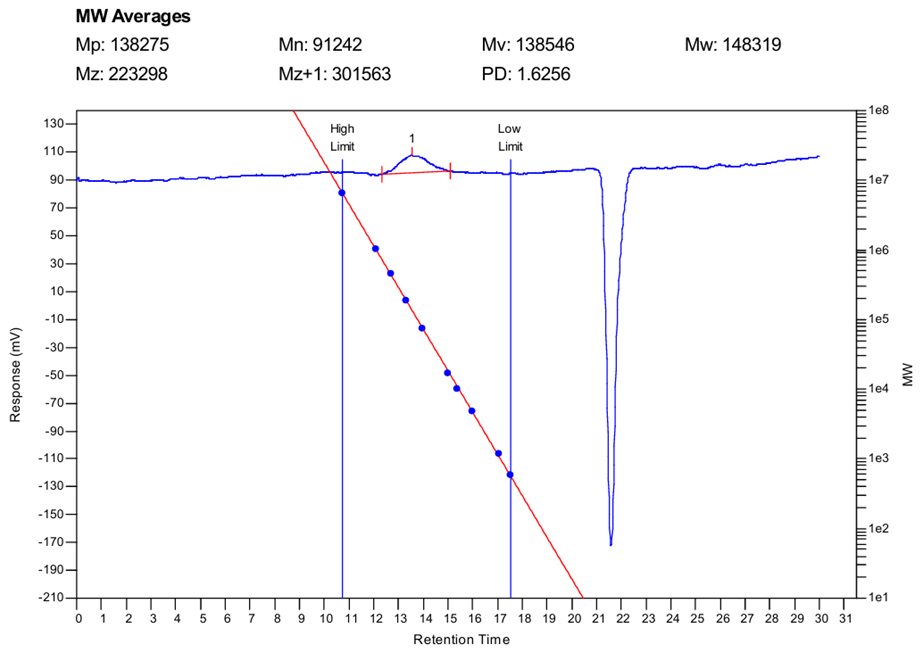


**Figure S17.** HT-GPC diagram of D18.


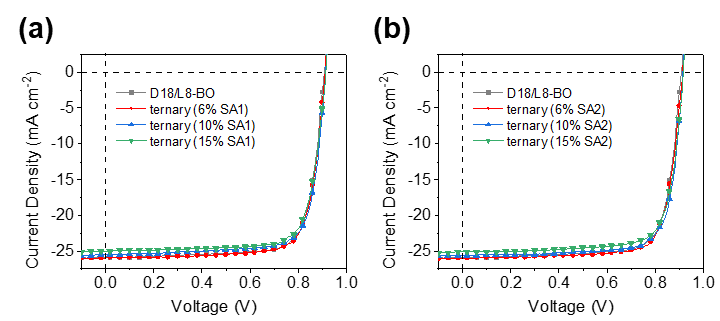


**Figure S18.** a, b) *J-V* curves of ternary devices based on SA1 (a) and SA2 (b).


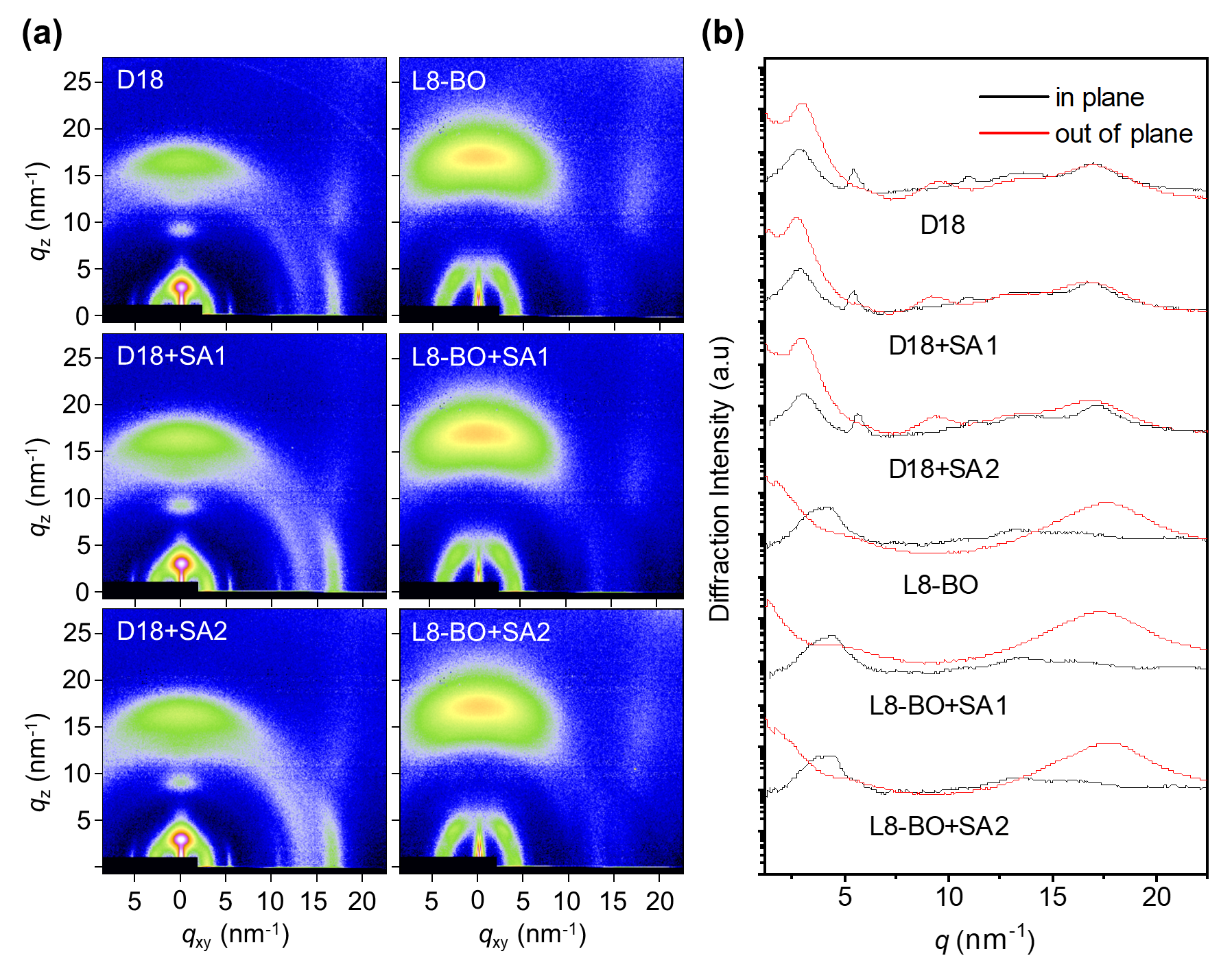


**Figure S19.** a) GIWAXS image of the thin films. b) In-plane and out-of-plane linecuts from (a).


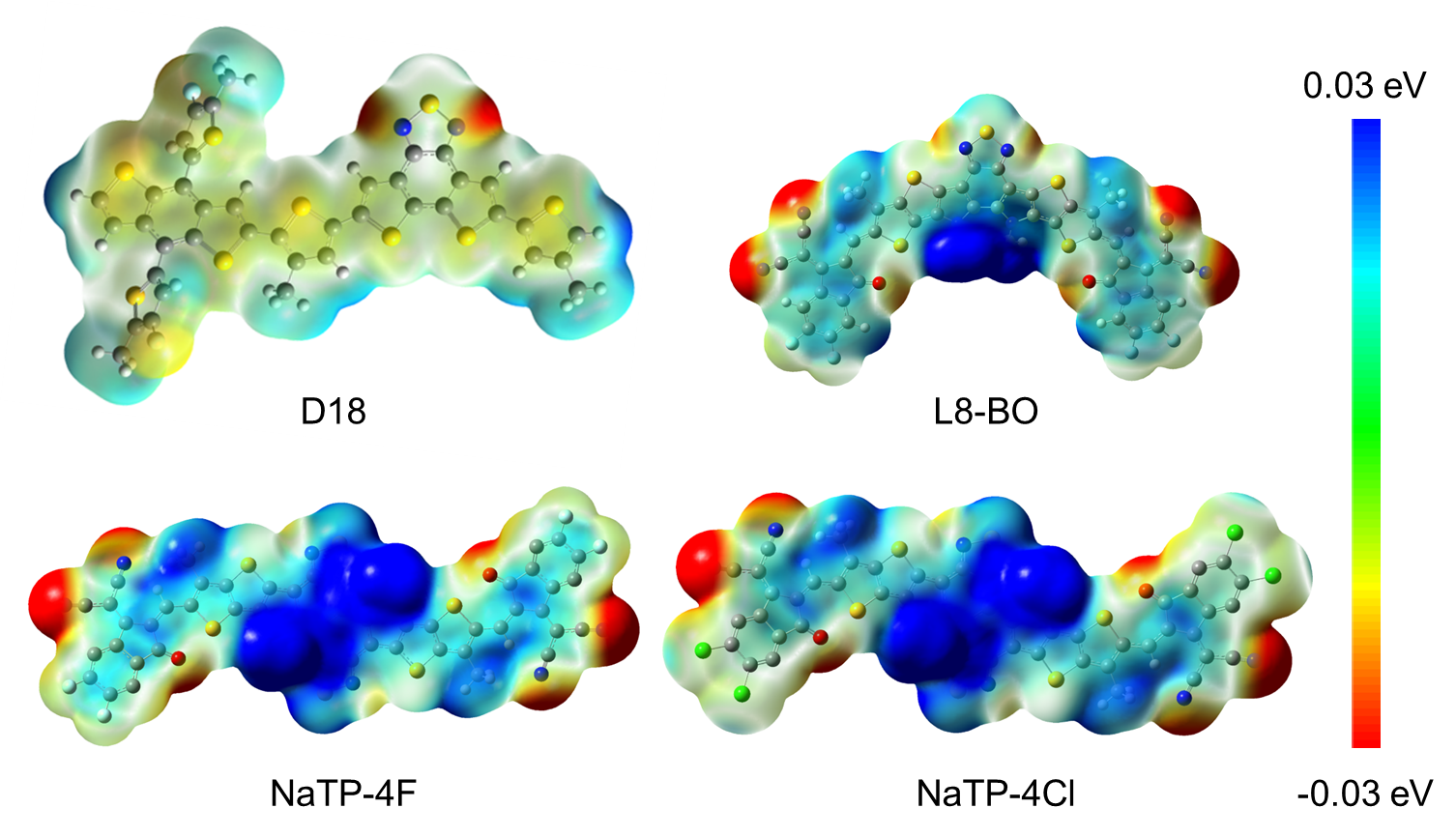


**Figure S20.** ESP distribution of D18, L8-BO and multifunctional solid additives.


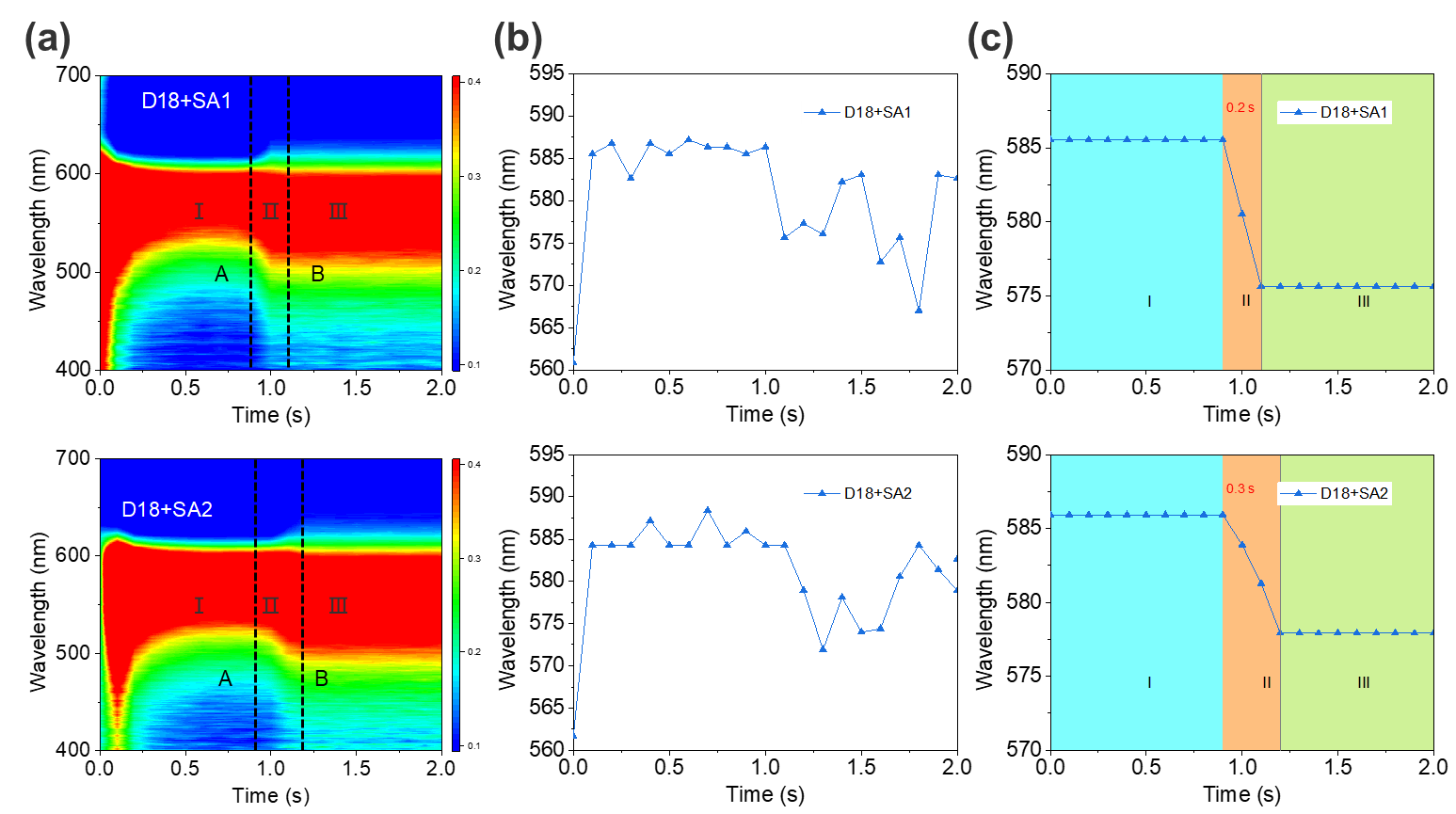


**Figure S21. a)** Time-dependent contour maps of in situ UV-vis absorption spectra of D18+SA1 and D18+SA2 films during spin coating. b) Extract the wavelength-time curves from (a). c) Time evolution of peak location.


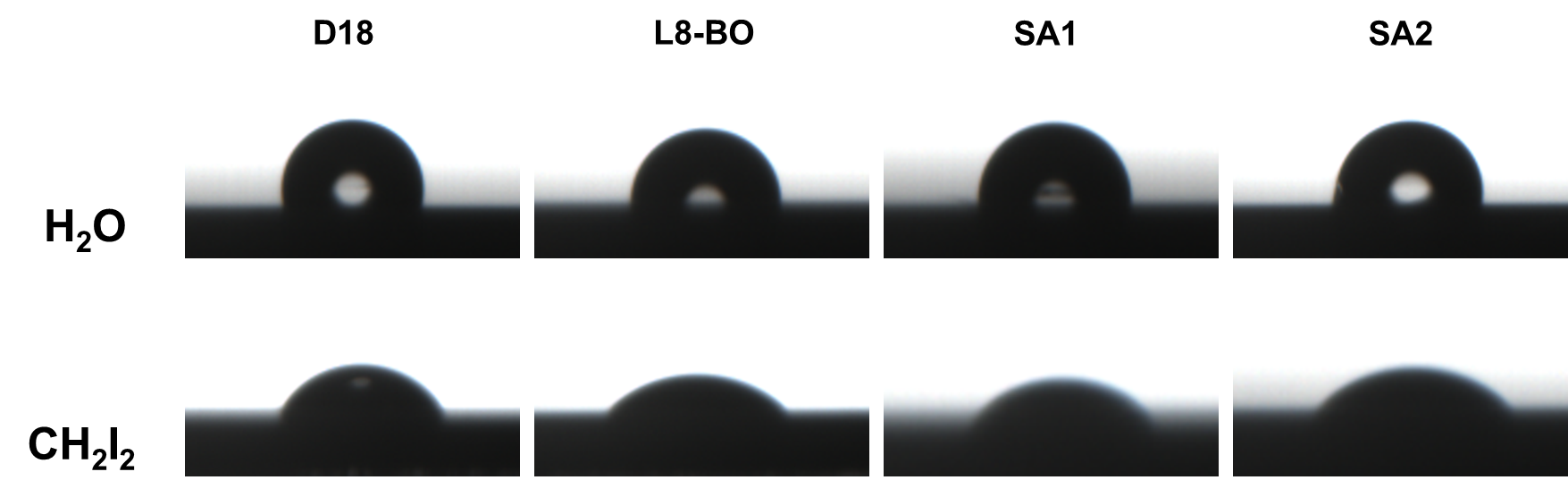


**Figure S22.** Surface energy measurements of D18, SA1, SA2 and L8-BO.


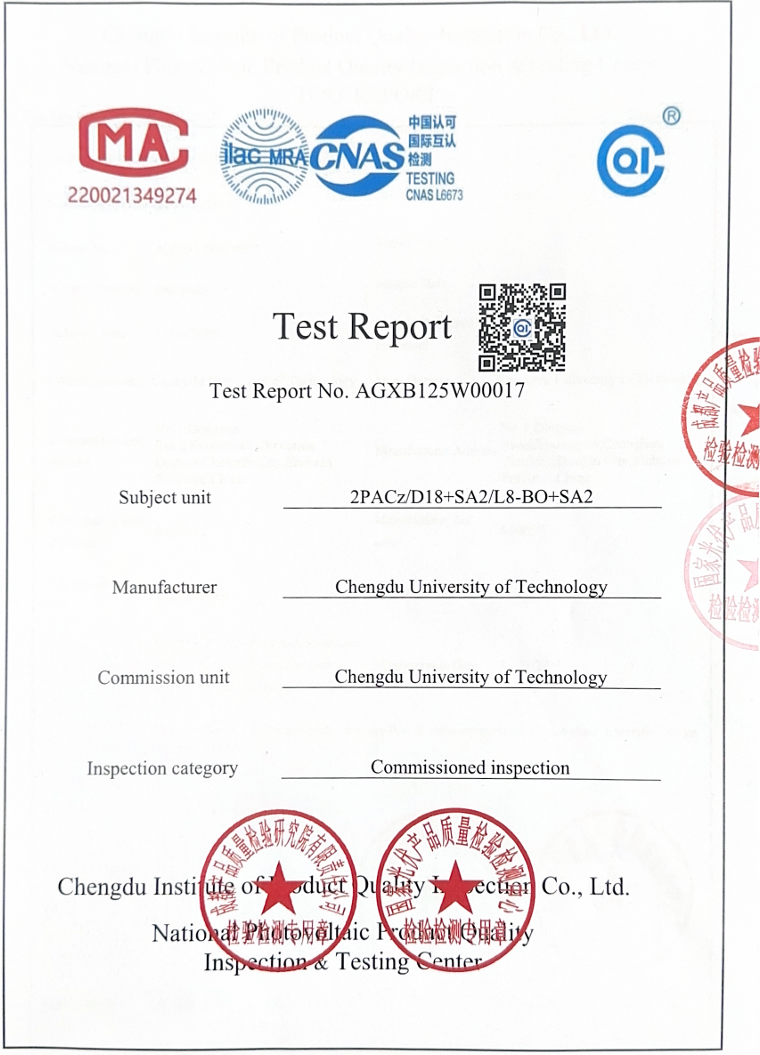


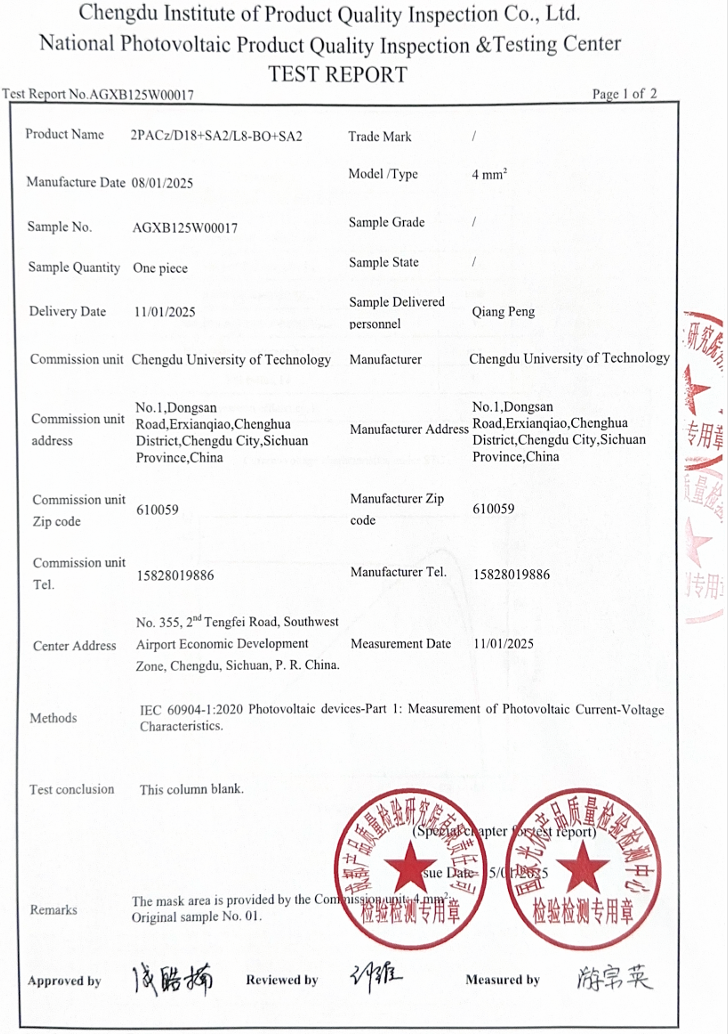


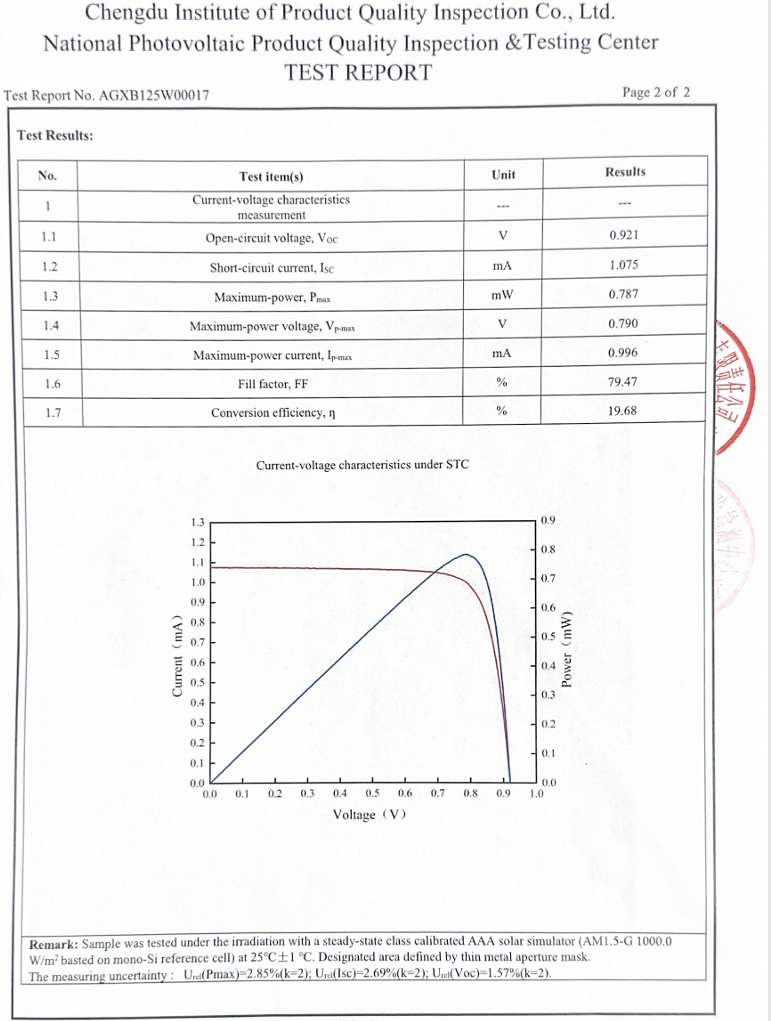


**Figure S23.** Certification report of D18+SA2/L8-BO+SA2 devices from Chengdu Institute of Product Quality Inspection Co., Ltd. National Photovoltaic Product Quality Inspection & Testing Center.


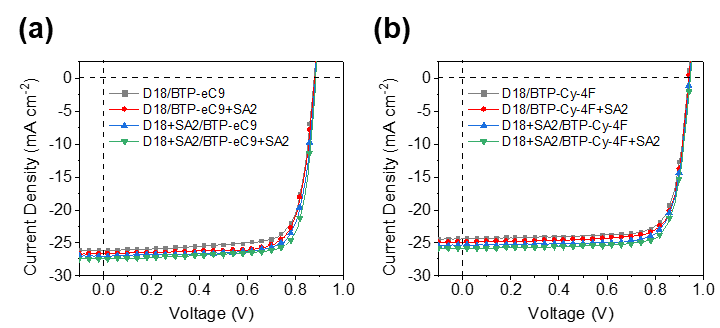


**Figure S24.** a, b) *J-V* curves of binary devices of D18/BTP-eC9 (a) and D18/BTP-Cy-4F with SA2 as the solid additives.


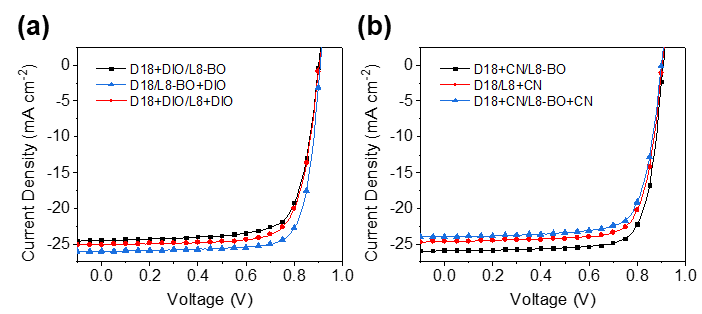


**Figure S25.** a, b) *J-V* curves of binary devices based on DIO (a) and CN (b) as the additives.

**Figure S26.** The stability curves of the optimal binary devices based on DIO, CN, SA1 and SA2 as the additives in a dry nitrogen atmosphere with encapsulation.


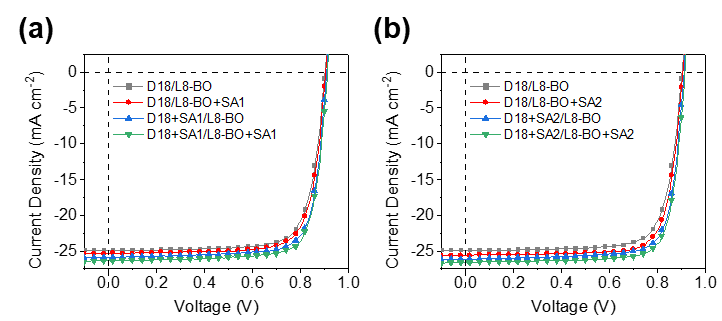


**Figure S27.** The device performance of the device by using PEDOT:PSS as hole-transporting layer.


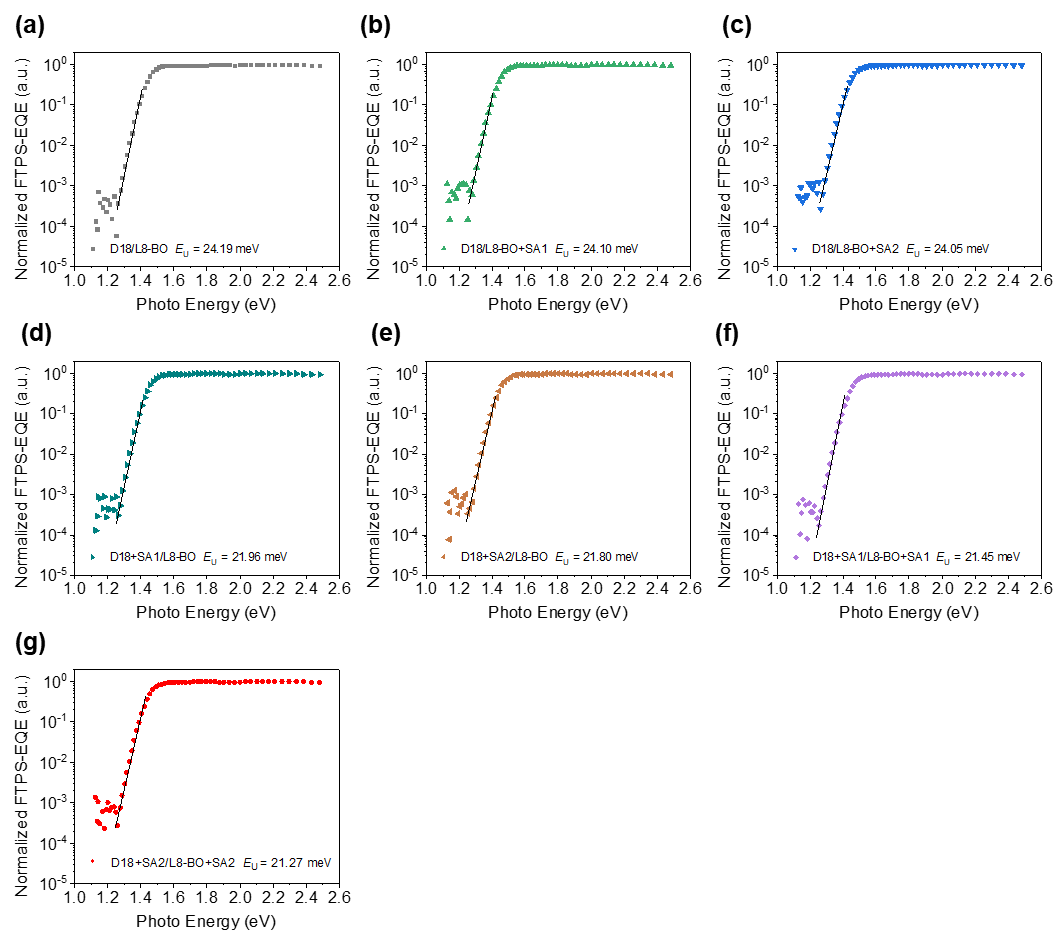


**Figure S28.** The full spectra of FTPS-EQE spectra and the corresponding fitting curves.


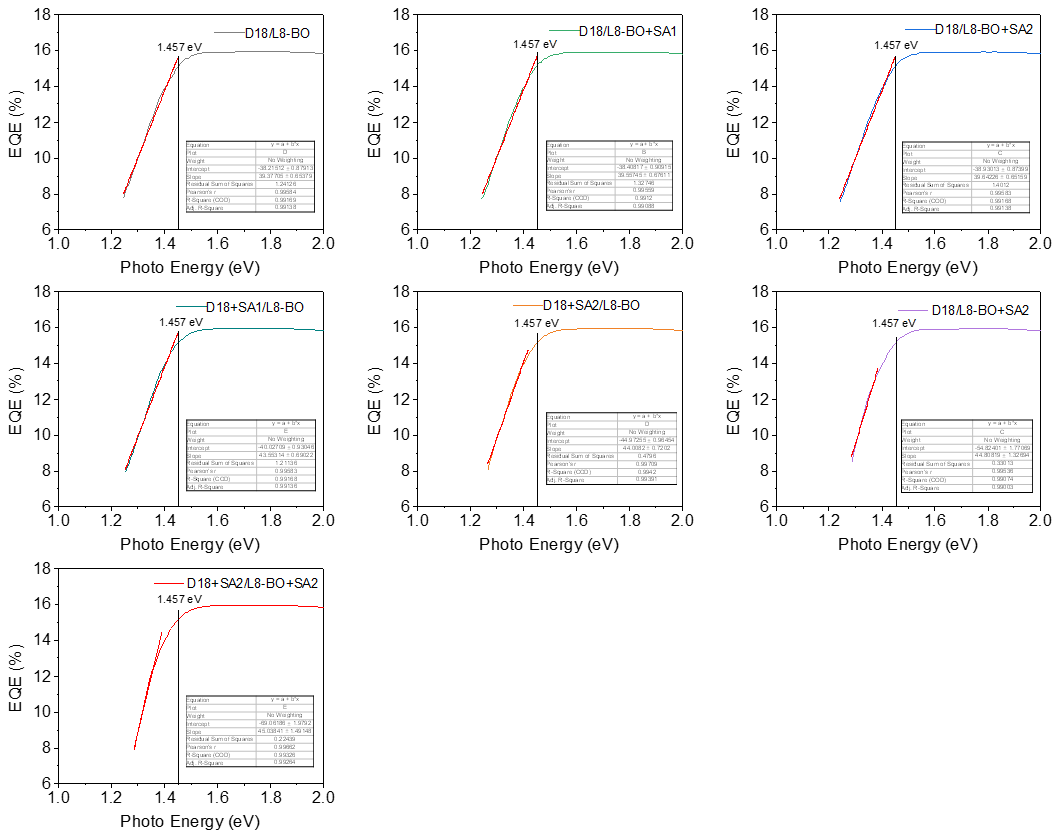


**Figure S29.** The fitting curves of the tail state absorption of the lnEQE curves.


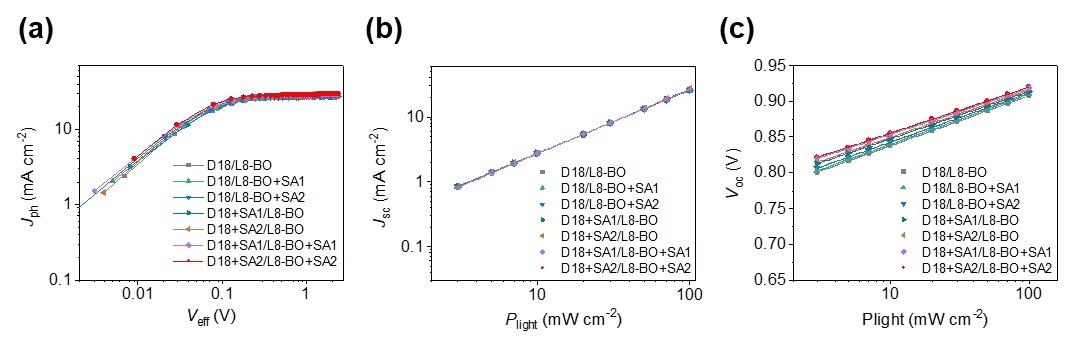


**Figure S30.** a) *J*_ph_ versus *V*_eff_ of the top-performing binary devices. b) *J*_sc_ versus *P*_light_ plots and (c) *V*_oc_ versus *P*_light_ plots of the top-performing binary devices.


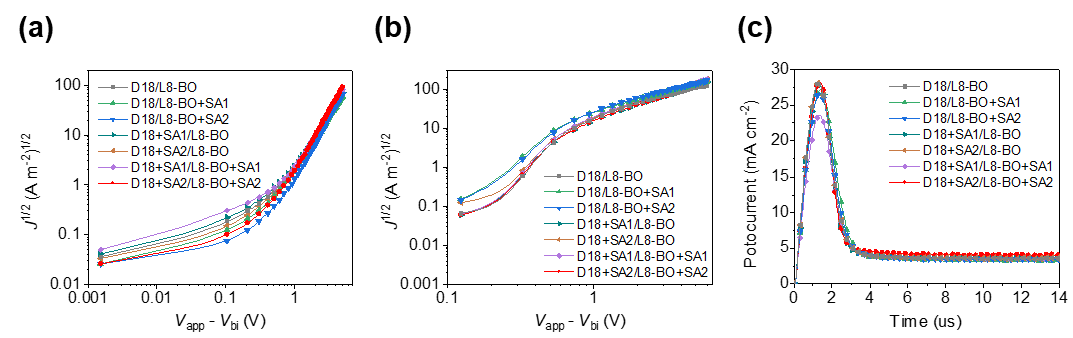


**Figure S31.** a) Hole mobilities and (b) electron mobilities of the PSCs. c) photo-CELIV curves of the PSCs.


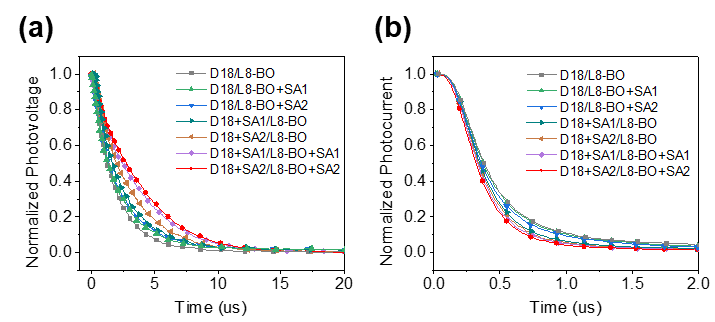


**Figure S32.** a) The transient photovoltage (TPV) and (b) transient photocurrent (TPC) of the PSCs.

**5. Supplementary Tables**

**Table S1.** Photovoltaic parameters of the binary devices based on D18/SA1 and D18/SA2.

| active layer | *V*_oc_  (V) | *J*_sc_  (mA cm^-2^) | *J*_sc_^EQE^  (mA cm^-2^) | FF  (%) | PCE  (%) |
| --- | --- | --- | --- | --- | --- |
| D18/SA1 | 0.969 | 20.03 | 19.10 | 65.55 | 12.72 |
| D18/SA2 | 0.937 | 21.92 | 20.88 | 69.79 | 14.33 |

**Table S2.** Photovoltaic parameters of the ternary devices based on SA1.

| acceptor | *V*_oc_  (V) | *J*_sc_  (mA cm^-2^) | FF  (%) | PCE  (%) |
| --- | --- | --- | --- | --- |
| L8-BO | 0.907 | 25.86 | 77.51 | 18.18 |
| L8-BO:SA1  (0.94:0.06) | 0.911 | 25.95 | 77.91 | 18.42 |
| L8-BO:SA1  (0.90:0.10) | 0.913 | 25.53 | 77.31 | 18.02 |
| L8-BO:SA1  (0.85:0.15) | 0.914 | 25.01 | 77.08 | 17.62 |

**Table S3.** Photovoltaic parameters of the ternary devices based on SA2.

| acceptor | *V*_oc_  (V) | *J*_sc_  (mA cm^-2^) | FF  (%) | PCE  (%) |
| --- | --- | --- | --- | --- |
| L8-BO | 0.907 | 25.86 | 77.51 | 18.18 |
| L8-BO:SA2  (0.94:0.06) | 0.913 | 26.05 | 78.12 | 18.58 |
| L8-BO:SA2  (0.90:0.10) | 0.915 | 25.61 | 77.62 | 18.19 |
| L8-BO:SA2  (0.85:0.15) | 0.917 | 25.17 | 77.28 | 17.84 |

**Table S4.** Summarized parameters for the ordered structures.

|  | Lamellar stacking | | | |  | π – π stacking | | | |
| --- | --- | --- | --- | --- | --- | --- | --- | --- | --- |
|  | *q*  (nm^-1^) | *d*  (nm) | FWHM  (nm^-1^) | CCL  (nm) |  | *q*  (nm^-1^) | *d*  (nm) | FWHM  (nm^-1^) | CCL  (nm) |
| D18 | 2.84 | 2.21 | 0.91 | 6.15 |  | 16.90 | 0.37 | 2.28 | 2.45 |
|  |  |  |  |  |  |  |  |  |  |
| D18+SA1 | 2.87 | 2.19 | 0.86 | 6.51 |  | 16.95 | 0.37 | 2.15 | 2.60 |
|  |  |  |  |  |  |  |  |  |  |
| D18+SA2 | 2.91 | 2.16 | 0.81 | 6.91 |  | 17.05 | 0.37 | 2.02 | 2.77 |
|  |  |  |  |  |  |  |  |  |  |
| L8-BO | 3.78 | 1.66 | 1.39 | 4.03 |  | 17.63 | 0.36 | 2.47 | 2.26 |
|  |  |  |  |  |  |  |  |  |  |
| L8-BO+SA1 | 3.76 | 1.67 | 1.40 | 4.00 |  | 17.65 | 0.36 | 2.48 | 2.25 |
|  |  |  |  |  |  |  |  |  |  |
| L8-BO+SA2 | 3.75 | 1.67 | 1.40 | 4.00 |  | 17.65 | 0.36 | 2.50 | 2.24 |
|  |  |  |  |  |  |  |  |  |  |
| D18/L8-BO | 2.91^(a)^ | 2.16 | 0.57 | 9.82 |  | 17.23 | 0.36 | - | - |
|  | 3.72^(b)^ | 1.69 | 0.65 | 8.61 |  |  |  |  |  |
|  |  |  |  |  |  |  |  |  |  |
| D18/L8-BO+SA1 | 2.92^(a)^ | 2.15 | 0.57 | 9.82 |  | 17.29 | 0.36 | - | - |
|  | 3.73^(b)^ | 1.68 | 0.65 | 8.61 |  |  |  |  |  |
|  |  |  |  |  |  |  |  |  |  |
| D18/L8-BO+SA2 | 2.93^(a)^ | 2.14 | 0.57 | 9.82 |  | 17.32 | 0.36 | - | - |
|  | 3.74^(b)^ | 1.68 | 0.65 | 8.61 |  |  |  |  |  |
|  |  |  |  |  |  |  |  |  |  |
| D18+SA1/L8-BO | 2.95^(a)^ | 2.13 | 0.54 | 10.36 |  | 17.35 | 0.36 | - | - |
|  | 3.74^(b)^ | 1.68 | 0.65 | 8.61 |  |  |  |  |  |
|  |  |  |  |  |  |  |  |  |  |
| D18+SA2/L8-BO | 2.98^(a)^ | 2.11 | 0.53 | 10.56 |  | 17.38 | 0.36 | - | - |
|  | 3.74^(b)^ | 1.68 | 0.65 | 8.61 |  |  |  |  |  |
|  |  |  |  |  |  |  |  |  |  |
| D18+SA1/L8-BO+SA1 | 3.00^(a)^ | 2.09 | 0.54 | 10.36 |  | 17.42 | 0.36 | - | - |
|  | 3.74^(b)^ | 1.68 | 0.65 | 8.61 |  |  |  |  |  |
|  |  |  |  |  |  |  |  |  |  |
| D18+SA2/L8-BO+SA2 | 3.02^(a)^ | 2.08 | 0.53 | 10.56 |  | 17.45 | 0.36 | - | - |
|  | 3.74^(b)^ | 1.68 | 0.65 | 8.61 |  |  |  |  |  |

^(a)^ Scatterings from the donor; ^(b)^ Scatterings from the acceptor.

**Table S5.** Parameters of contact angles and surface energies of films.

| film | contact angle (^o^) | | surface energy (*γ*) (mJ m^-2^) | *χ*_D-A_ |
| --- | --- | --- | --- | --- |
|  | H_2_O | CH_2_I_2_ |  |  |
| D18 | 101.017 | 57.218 | 30.46 | 0.39 |
| L8-BO | 93.576 | 44.855 | 37.72 |  |
|  |  |  |  |  |
| D18 | 101.017 | 57.218 | 30.46 | 0.17 |
| SA1 | 90 | 51.34 | 35.24 |  |
|  |  |  |  |  |
| D18 | 101.017 | 57.218 | 30.46 | 0.19 |
| SA2 | 94.905 | 48.95 | 35.49 |  |
|  |  |  |  |  |
| SA1 | 90 | 51.34 | 35.24 | 0.04 |
| L8-BO | 93.576 | 44.855 | 37.72 |  |
|  |  |  |  |  |
| SA2 | 94.905 | 48.95 | 35.49 | 0.03 |
| L8-BO | 93.576 | 44.855 | 37.72 |  |

**Table S6.** Photovoltaic parameters of the D18/BTP-eC9 devices with SA2 as the solid additives.

| active layer | *V*_oc_  (V) | *J*_sc_  (mA cm^-2^) | FF  (%) | PCE  (%) |
| --- | --- | --- | --- | --- |
| D18/BTP-eC9 | 0.877 | 26.08 | 76.86 | 17.58 |
| D18/BTP-eC9+SA2 | 0.879 | 26.64 | 77.53 | 18.15 |
| D18+SA2/BTP-eC9 | 0.882 | 27.00 | 78.62 | 18.72 |
| D18+SA2/BTP-eC9+SA2 | 0.884 | 27.41 | 79.31 | 19.22 |

**Table S7.** Photovoltaic parameters of the D18/BTP-Cy-4F devices with SA2 as the solid additives.

| active layer | *V*_oc_  (V) | *J*_sc_  (mA cm^-2^) | FF  (%) | PCE  (%) |
| --- | --- | --- | --- | --- |
| D18/BTP-Cy-4F | 0.937 | 24.36 | 78.72 | 17.97 |
| D18/BTP-Cy-4F+SA2 | 0.939 | 24.87 | 79.02 | 18.45 |
| D18+SA2/BTP-Cy-4F | 0.942 | 25.33 | 79.52 | 18.97 |
| D18+SA2/BTP-Cy-4F+SA2 | 0.944 | 25.81 | 79.93 | 19.47 |

**Table S8.** Photovoltaic parameters of the D18/L8-BO devices with DIO as the additive.

| active layer | *V*_oc_  (V) | *J*_sc_  (mA cm^-2^) | FF  (%) | PCE  (%) |
| --- | --- | --- | --- | --- |
| D18+DIO/L8-BO | 0.901 | 24.52 | 74.35 | 16.43 |
| D18/L8-BO+DIO | 0.907 | 26.01 | 78.33 | 18.48 |
| D18+DIO/L8-BO+DIO | 0.903 | 25.08 | 75.23 | 17.04 |

**Table S9.** Photovoltaic parameters of the D18/L8-BO devices with CN as the additive.

| active layer | *V*_oc_  (V) | *J*_sc_  (mA cm^-2^) | FF  (%) | PCE  (%) |
| --- | --- | --- | --- | --- |
| D18+CN/L8-BO | 0.907 | 25.92 | 77.94 | 18.32 |
| D18/L8-BO+CN | 0.903 | 24.64 | 76.28 | 16.97 |
| D18+CN/L8-BO+CN | 0.900 | 24.00 | 75.49 | 16.31 |

**Table S10.** Photovoltaic parameters of using PEDOT:PSS as hole-transporting layer.

| active layer | *V*_oc_  (V) | *J*_sc_  (mA cm^-2^) | FF  (%) | PCE  (%) |
| --- | --- | --- | --- | --- |
| D18/L8-BO | 0.902 | 24.91 | 76.89 | 17.28 |
| D18/L8-BO+SA1 | 0.904 | 25.32 | 77.41 | 17.72 |
| D18/L8-BO+SA2 | 0.905 | 25.59 | 77.78 | 18.01 |
| D18+SA1/L8-BO | 0.908 | 25.87 | 78.16 | 18.36 |
| D18+SA2/L8-BO | 0.910 | 26.12 | 78.41 | 18.64 |
| D18+SA1/L8-BO+SA1 | 0.912 | 26.36 | 78.93 | 18.98 |
| D18+SA2/L8-BO+SA2 | 0.914 | 26.62 | 79.15 | 19.26 |

**Table S11.** Energy loss of the PSCs.

| active layer | *E*_g_  (eV) | EQE_EL_@*J*_SC_ | *V*_oc, SQ_  (V) | *V*_oc, rad_  (V) | Δ*E*_1_  (eV) | Δ*E*_2_  (eV) | Δ*E*_3_  (eV) | *E*_loss_  (eV) |
| --- | --- | --- | --- | --- | --- | --- | --- | --- |
| D18/L8-BO | 1.457 | 0.025% | 1.177 | 1.115 | 0.280 | 0.062 | 0.213 | 0.555 |
| D18/L8-BO+SA1 | 1.457 | 0.026% | 1.177 | 1.116 | 0.280 | 0.061 | 0.211 | 0.552 |
| D18/L8-BO+SA2 | 1.457 | 0.028% | 1.177 | 1.117 | 0.280 | 0.060 | 0.209 | 0.549 |
| D18+SA1/L8-BO | 1.457 | 0.032% | 1.178 | 1.119 | 0.279 | 0.059 | 0.207 | 0.545 |
| D18+SA2/L8-BO | 1.457 | 0.034% | 1.178 | 1.120 | 0.279 | 0.058 | 0.205 | 0.542 |
| D18+SA1/L8-BO+SA1 | 1.457 | 0.039% | 1.179 | 1.123 | 0.278 | 0.056 | 0.202 | 0.536 |
| D18+SA2/L8-BO+SA2 | 1.457 | 0.044% | 1.179 | 1.124 | 0.278 | 0.055 | 0.199 | 0.532 |

**6. References**

[1] M. Wang, X. Hu, P. Liu, W. Li, X. Gong, F. Huang, Y. Cao, *J. Am. Chem. Soc.* **2011**, *133*, 9638-9641.

[2] J. Yuan, Y. Zhang, L. Zhou, G. Zhang, H. L. Yip, T. K. Lau, X. Lu, C. Zhu, H. Peng, P. A. Johnson, M. Leclerc, Y. Cao, J. Ulanski, Y. Li, Y. Zou, *Joule* **2019**, *3*, 1140-1151.

[3] C. Kaiser, O. J. Sandberg, N. Zarrabi, W. Li, P. Meredith, A. Armin, *Nat. Commun.* **2021**, *12*, 3988.

[4] C. Zhang, S. Mahadevan, J. Yuan, J. K. W. Ho, Y. Gao, W. Liu, H. Zhong, H. Yan, Y. Zou, S. W. Tsang, S. K. So, *ACS Energy Lett.* **2022**, *7*, 1971-1979.

[5] K. Vandewal, K. Tvingstedt, A. Gadisa, O. Inganäs, J. V. Manca, *Nat. Mater.* **2009**, *8*, 904-909.

[6] U. Rau, *Phys. Rev. B* **2007**, *76*, 085303.

[7] W. Shockley, H. J. Queisser, *J. Appl. Phys.***1961**, **32**, 510.
